# Using Evidence-Based Internet Interventions to Reduce Health Disparities Worldwide

**DOI:** 10.2196/jmir.1463

**Published:** 2010-12-17

**Authors:** Ricardo F Muñoz

**Affiliations:** ^3^Internet World Health Research CenterUniversity of CaliforniaSan Francisco, CAUSA; ^2^Latino Mental Health Research ProgramUniversity of CaliforniaSan Francisco, CAUSA; ^1^Department of Psychiatry at San Francisco General HospitalUniversity of CaliforniaSan Francisco, CAUSA

**Keywords:** Internet, online treatment, prevention, clinical trials, health disparities, consumable interventions, smoking, depression, public health, evidence-based, Internet interventions

## Abstract

Health disparities are a persistent problem worldwide. A major obstacle to reducing health disparities is reliance on “consumable interventions,” that is, interventions that, once used, cannot be used again. To reduce health disparities, interventions are required that can be used again and again without losing their therapeutic power, that can reach people even if local health care systems do not provide them with needed health care, and that can be shared globally without taking resources away from the populations where the interventions were developed.

This paper presents the argument that automated self-help evidence-based Internet interventions meet the above criteria and can contribute to the reduction of health disparities worldwide. Proof-of-concept studies show that evidence-based Internet interventions can reach hundreds of thousands of people worldwide and could be used in public sector settings to augment existing offerings and provide services not currently available (such as prevention interventions). This paper presents a framework for systematically filling in a matrix composed of columns representing common health problems and rows representing languages.

To bring the benefits of evidence-based Internet interventions to the underserved, public sector clinics should establish eHealth resource centers, through which patients could be screened online for common disorders and provided with evidence-based Internet intervention services not currently available at the clinics. These resources should be available in the patients’ languages, in formats that do not require literacy, and that can be accessed with mobile devices. Such evidence-based Internet interventions should then be shared with public sector clinics as well as individuals anywhere in the world.

Finally, this paper addresses sustainability and describes a continuum of evidence-based Internet interventions to share nationally and across the world. This approach to expanding health service delivery will significantly contribute to a reduction of health disparities worldwide, adding to the often-quoted slogan, “Think globally, act locally,” a third line: “Share globally.”

## Introduction

### A Personal Comment

The author’s commitment to using the Internet to help reduce disparities worldwide stems in part from his personal background. He grew up in a small community named Chosica, 40 kilometers east of Lima, Peru. When he was ten, his mother (Clara Luz Valdivia de Muñoz) informed him that the family was going to immigrate to the United States of America. His father (Luis Alberto Muñoz Camino) had a primary school education, his mother had completed high school, and they now wanted their children to attend university. Not being able to afford such in Peru, they were seeking the educational and economic opportunities available in the United States. The plan, his mother said, was for them to return to Peru after obtaining a university education and share their knowledge with the people in Peru. As is often the case with immigrants, they did not return to live in their country of origin. But the mission she instilled in the author of sharing knowledge with as many people as possible remains an essential part of his motivation as a professional.

There are thousands of communities like Chosica throughout the world. The Internet provides an efficient conduit to convey to such communities the most effective preventive and treatment behavioral interventions in the form of evidence-based Internet interventions [1,2].

### Objective

Described below are 5 types of Internet interventions. The least costly to sustain are automated self-help Internet interventions (without additional guidance by online, phone, or in-person providers). The author’s experience conducting randomized controlled trials of such interventions with individuals from anywhere in the world has provided proof of concept regarding the effectiveness of such interventions for smoking cessation. This paper builds upon this experience and asks the reader to imagine how the use of evidence-based Internet interventions for this and other health problems could contribute to the reduction of one aspect of health disparities, namely, having inadequate or no access to evidence-based interventions.

### Reliance on Consumable Interventions Limits Access to Health Care

There are over 1.1 billion smokers [3], over 121 million people with clinical depression [4], and over 76 million people with alcohol use disorders [5]. We will never train enough health care providers to administer adequate health care for all who need it if we continue our reliance on “consumable interventions,” that is, interventions that, once used, cannot be used again. For example, a dose of medication can only be used once; the time spent treating a patient cannot be used ever again to treat another patient.

To reduce health disparities, we need interventions that can be used again and again without losing their therapeutic power, that can reach people even if local health care systems cannot or will not provide them with needed health care, and that can be shared widely without taking resources away from the populations where the interventions were developed.

Evidence-based Internet interventions meet all these qualifications. Evidence-based Internet interventions are empirically tested online methods to change individual behavior to prevent or ameliorate health problems. Medications are the active ingredient in many medical interventions; information in the form of “behavioral prescriptions” is the active ingredient in Internet interventions. Both can be standardized, evaluated using randomized controlled trials, and disseminated globally as long as users have funds to buy the medication or access the Web. Both can be used cross-culturally, as long as they are provided in a linguistically and culturally acceptable manner.

A major advantage of fully automated self-help Internet interventions is cost. Bringing a medication to market costs hundreds of millions of dollars [6] while developing an Internet intervention may cost as little as a few thousand dollars for a simple site, or, at most, several hundreds of thousands of dollars. Costs of testing a site in randomized controlled trials may run in the hundreds of thousands of dollars but, in general, require many fewer dollars per trial participant than face-to-face interventions. The differences in per-person costs of administering the intervention are even more impressive: a dose of a medication has a minimal cost even when bought in large quantities; the marginal cost of providing a self-help automated Internet intervention to one additional user eventually approaches zero. The cost of hosting the site remains a steady expense, of course, but, after the server has made the site available to say, 50,000 users, the cost of serving one more user becomes negligible. This makes it possible to share the site with people worldwide, without taking anything away from the communities where the intervention was developed.

## Imagine: How Evidence-Based Internet Interventions Could Contribute to Reducing Health Disparities

### Transcending Space and Time

There are health interventions that can transcend space. Traditional telemedicine consultation, for example, can connect a specialist with a primary care provider and a patient anywhere in the world. However, the time spent with a patient in another locale is time that is not being used to serve patients locally. Internet interventions transcend both space and time: they can be used simultaneously anywhere in the world, and they can be used again and again, at a time of the person’s choosing, even if the specialists who developed and tested the site are no longer active professionally or even no longer alive. In this sense, Internet interventions transcend both space and time.

### How Public Sector Clinics Could Use Evidence-Based Internet Interventions 

Internet interventions could help extend health services to patients who are experiencing disparities in terms of care. Imagine that a Spanish-speaking patient who does not speak English arrives at San Francisco General Hospital. She is provided with a mobile device or directed to an eHealth resource room to be screened in her own language for the most common health problems (ideally using audio and video, should she not be able to read). The results would be printed in her own language and her providers’ language (in this case, Spanish and English). If she meets criteria for major depressive episode, her physician might prescribe antidepressants and recommend cognitive-behavioral therapy for depression. If there are no cognitive-behavioral therapists who speak Spanish available, the patient could receive a cognitive-behavioral Internet intervention for depression in Spanish either at the eHealth resource room or, preferably, via a mobile device she could take home. Once such an intervention has been developed and tested at San Francisco General Hospital, it could be shared via the Web with any clinic anywhere in the world. A similar vignette could be described with a Quechua-speaking person going to a public sector clinic in Lima, Peru, or a Laotian-speaking patient at an Amsterdam clinic.

Live health care providers should not be replaced by Internet interventions, even evidence-based Internet interventions. Doing so would be a travesty. Each person who needs health care anywhere in the world should ideally be provided with a physician, nurse, psychologist, and so on, who are well trained to provide evidence-based interventions. Until this has been achieved, we should provide people with some intervention that is effective rather than not providing anything. Evidence-based Internet interventions are, at minimum, a temporary stopgap measure to provide needed interventions where none are available now. However, it will likely take many lifetimes, if ever, before adequate health care becomes accessible to all. Thus, efforts aimed at developing, testing, and disseminating nontraditional interventions to expand health services delivery are a good use of professional time and are sure to benefit large numbers of people [7]. Studying the comparative effectiveness of these interventions, including Internet interventions, for specific health problems in specific populations, would bring empirical evidence to bear on how best to utilize such interventions.

### Addressing Unmet Needs

Internet interventions can be used:

- When no other interventions for specific health problems are available
- While patients are on waiting lists, for example, during the weeks between being referred for treatment and the time a slot is open for a smoking cessation group or a depression group
- During routine treatment, as an adjunct, for example, offering an Internet cognitive-behavioral intervention as an adjunct to pharmacotherapy for depression offered in a primary care clinic
- After treatment, to prevent relapse or recurrence
- For patients who cannot travel to clinics due to distance, physical limitations, time limitations, or economic limitations
- For patients who fear stigma if they come to a mental health clinic
- For patients whose providers do not speak their language
- To extend health care beyond treatment into prevention

### Beyond Treatment: Providing Preventive Interventions

The Institute of Medicine (IOM) of the United States of America has published two reports on the prevention of mental, emotional, and behavioral disorders; one, in 1994 [8] and the second, in 2009 [9]. These reports call for changes in health care policy to include prevention as a routine offering. Preventive interventions have been shown to reduce the incidence (that is, the number of new cases) of mental, emotional, and behavioral disorders. Health care systems currently spend most of their resources on treatment. They do this to reduce the prevalence of disorders, that is, the total number of cases of illness. However, one way to reduce prevalence is to reduce incidence, that is, the number of new cases. To reduce incidence, effective preventive programs must be provided. Health care administrators are in a dilemma. Facing limited budgets, they often find it hard to channel funds into prevention when they feel they are not providing adequate treatment services. Could Internet interventions help?

**Figure 1:**
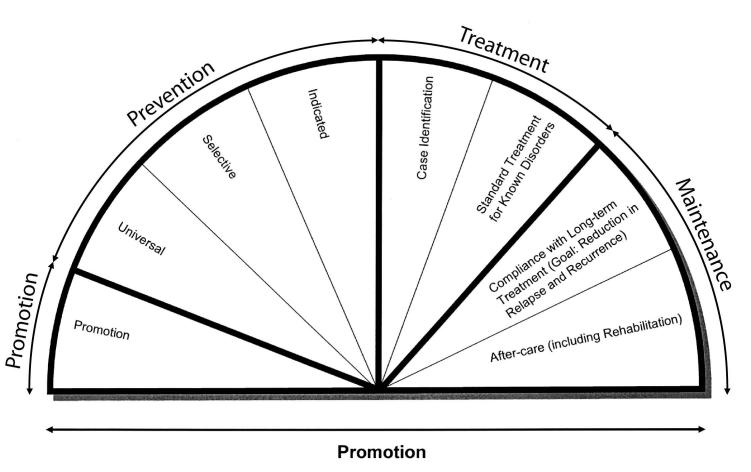
The mental health intervention spectrum [9] (Reprinted with permission from Preventing Mental, Emotional, and Behavioral Disorders among Young People: Progress and Possibilities, 2009, National Academy of Sciences, by the courtesy of the National Academies Press, Washington, DC.)

The 1994 IOM report argued that it is important to define prevention as interventions that occur prior to the onset of the targeted disorder (see Figure 1). Individuals with the disorder need to be identified and provided treatment. Once the acute phase of the disorder has abated, maintenance interventions should be provided to prevent relapse or recurrence or to provide rehabilitation services to reduce the sequelae of the acute episode. Prevention is itself divided into 3 levels: *universal* interventions for entire populations, *selective* interventions for subgroups at higher risk because of characteristics that are known to increase incidence (eg, poverty, trauma, or loss of a loved one), and *indicated* interventions for individuals showing early signs or symptoms of the targeted disorder but not yet meeting diagnostic criteria.

Let’s look at a specific disorder. There are now many studies showing that prevention interventions can significantly reduce the number of new cases of major depression [10]. Yet, these interventions are not routinely offered. Even where preventive interventions have been developed and tested, once research funding ends, the institution generally does not fund ongoing prevention services. However, if the preventive intervention were an Internet intervention, maintaining the site online would not only provide access to the intervention (as tested) for the local setting, but for thousands or even hundreds of thousands of people who would otherwise not receive the benefit of preventive interventions.

### Are Evidence-Based Internet Interventions Effective in Real World Settings?

The argument presented thus far assumes that Internet interventions can be effective. Is there evidence for such a claim? Isaac Marks and colleagues have reviewed an impressive collection of studies that support the contention that computer-assisted therapeutic interventions can, in fact, be effective [11,12]. Formal meta-analyses of the literature have also shown evidence of effectiveness for many health problems [13,14]. More recent reports continue to show empirical support for the effectiveness of Internet interventions [15,16] and their potential for both prevention and treatment [17,18]. Several articles have addressed the scientific foundation of Internet interventions [19-24].

The Latino Mental Health Research Program of the University of California, San Francisco, at San Francisco General Hospital, began work in this area in 1998. Until then, we had been developing and testing face-to-face individual and group interventions for the prevention and treatment of depression and for smoking cessation in English and Spanish, while some had been developed in Chinese as well. Our manuals, designed to train and help providers administer prevention and treatment interventions with fidelity to the intervention protocol as designed, are available at the UCSF/SFGH Latino Mental Health Research Program website [25] at no charge to anyone who wants to use them. We have had thousands of downloads of these materials. They are used in several countries and have been tested in randomized control trials in many settings with success [1]. However, a health care provider is needed to administer these interventions. Even where there are providers, clinicians tend to “drift” away from the treatment protocol due to time constraints, theoretical predilections, lack of experience, or simple human error [26]. Thus, even manualized interventions are often not provided as tested in randomized control trials.

Concerns that many communities have no access to trained professionals to administer our manuals prompted our group to begin experimenting with creating automated self-help Internet interventions and testing them in randomized control trials conducted on the Web. Thus, the intervention is tested exactly as it will be provided after the trial ends. This eliminates the problem of “drift.” In addition, the intervention is designed to be used directly by the individual and does not require the availability of a health care provider. Nor does this approach require the difficult negotiations involved in trying to convince regional, national, or international health care systems to institute empirically supported services.

We began our research on evidence-based Internet interventions with smoking cessation interventions to determine whether we could “match the patch,” that is, obtain abstinence rates comparable to those found for the nicotine patch. Randomized trials comparing placebo patches with nicotine patches have found 6-month quit rates of 5% to 8% for placebo patches and 14% to 22% for nicotine patches [27]. Because of the well-known problem with attrition in Internet studies [28], we used the conservative “missing = smoking” convention in which a participant who does not provide data is assumed to be smoking. Using this convention, we have obtained 6-month quit rates as high as 26% [2] and 12-month quit rates of 20% [29]. Our trials thus far have involved over 60,000 consenting smokers from over 200 countries (for a report on the reach of one of our trials, see [30]). But well over 600,000 visitors have come to the site, where they can download our Guide to Stop Smoking without having to join the study. We have been able to recruit people worldwide with the help of a Google grant that allows us to post “sponsored links” (ie, Google ads) on its search engine, in English and in Spanish, all over the world.

## A Proposal to Create a Central Exchange for Evidence-Based Internet Interventions

Our smoking cessation trials are proof of concept for how evidence-based Internet interventions can provide health interventions worldwide. The Latino Mental Health Research Program established the University of California, San Francisco/San Francisco General Hospital Internet World Health Research Center in 2004. Our goal is to systematically develop and test evidence-based Internet interventions to fill in a matrix of evidence-based Internet interventions for health problems by languages (see Table 1).

**Table 1:** Matrix for systematic development of evidence-based Web interventions: health problems by languages (our goal is to fill this matrix)

	Smoking	Depression	Pain	Diabetes	Obesity	Additional Health Problems
English						
Spanish						
Chinese						
Arabic						
Portuguese						
Additional languages						

This grid is infinitely expandable and will require the contributions of many health experts for the foreseeable future. A third dimension to the grid could represent specific subpopulations (men and women, young and old, diverse ethnic groups, preliterate and well-educated, rich and poor, and so on).

Once evidence-based Internet interventions become more numerous, we will need a system to help users choose among the many interventions that will populate the Web. The author proposes that a credible international body, such as the World Health Organization, create a Website to function as a central exchange or clearinghouse for empirical information on evidence-based Internet intervention sites. A site already exists, called “Beacon” [31], that lists Internet intervention sites and rates them on their empirical support. To expand upon this idea, a central evidence-based Internet interventions website could itself conduct randomized control trials of Internet interventions submitted to the clearinghouse. This would standardize recruitment procedures, administration of the interventions, and outcome assessments. The central evidence-based Internet intervention website would then provide a “box score” on the results. Users would be able to enter demographic data (eg, sex, age, language, and educational level) and receive outcome data for that subset of participants and the number of individuals on whom these data are based. Cost for use of commercial sites would be included, so public and commercial sites could be compared head to head.

Users could search for Internet interventions for depression, for example, and find the box score site for their demographic profile (See Table 2). Users could then decide whether the public site (Site B) would be worth trying. If they did so and site B did not help them, and if they chose to pay the price ($49), they could try site A. However, they may decide that site C might not be a good use of resources because it is both relatively expensive ($250) and its outcomes are inferior to sites A and B.

**Table 2:** Simplified illustration of how a central website for evidence-based Internet interventions might present data for a specific demographic profile

Website Intervention to Manage Depression	No Longer Depressed	Noticeably Improved, but Not Back to Normal Mood	Cost
A	52%	30%	$49
B	36%	54%	Public access
C	22%	20%	$250

## Discussion

### Sustainability

To reduce health disparities, evidence-based Internet interventions should be available worldwide at no charge. This would encourage public health clinics to maintain eHealth resource rooms for low- or no-income patients. It would also provide users who are unable to pay and who have no access to clinics with access to the evidence-based Internet interventions. The bulk of the cost of self-help automated Internet interventions is in their development and the research costs of testing them. Once found effective, keeping these Internet interventions active online requires a relatively modest expenditure. Nevertheless, the cost of maintaining these sites is not zero. Staff is needed to monitor emails from users who request help in using the site or send complaints or thank-you messages and to host and troubleshoot the site. Unless a major donor or global health organization provides ongoing funding for such sites, developers will need to create a revenue stream to maintain them.

One possibility would be to use evidence-based Internet interventions themselves to generate revenue and then funnel the revenue into the creation and maintenance of additional sites for other health problems and languages. For example, an evidence-based Internet intervention found to be effective for smoking cessation could be licensed to a health maintenance organization or a multinational corporation so the members or employees of these organizations could have special access to the site. The funds from the license could be used to maintain both the private site and a public version of the site.

### Commercial versus Public Access Evidence-Based Internet Interventions

Successful commercial sites are likely to have bigger budgets, faster development times, and a profit motivation to reach more people. Disadvantages of commercial sites include perceived conflict of interest in terms of providing honest reports of participants’ outcomes, similar to the concerns over pharmaceutical companies providing accurate and complete reports of the benefits and risks involved in using their products. There is also the danger that commercial sites may copyright or patent interventions that were formerly freely accessible, thus making them “proprietary.” Advantages of public access sites could include becoming known for their high quality in the same way that public television programming in the United States has achieved an excellent reputation [32]. Public access sites would reach those in need regardless of their ability to pay and regardless of the local health authorities’ ability or commitment to fund the sites. The outcome studies of such sites would be more credible, since financial conflict of interest would be less of an issue. Disadvantages of public access sites include the constant need to seek funding from donors, a slower pace in developing interventions, due in part to funding issues, and the possible perception of lower quality (ie, “you get what you pay for”).

### Five Levels of Internet Interventions 

Most health care providers aspire to a world in which everyone has access to needed health care. This would include access to well-trained providers who can administer evidence-based interventions, including preventive, treatment, and maintenance interventions. This ideal is unlikely to be achieved in the foreseeable future. But it is possible to begin to approach this ideal by systematically creating a continuum of health interventions that utilize evidence-based Internet interventions.

#### Level 1: Automated Self-help Internet Interventions 

These evidence-based Internet interventions are the most likely to contribute to the reduction of health disparities worldwide because they have the lowest ongoing cost. They consist of health behavior change interventions that are totally programmed. They can be individualized to as great an extent as programming allows, including sending individually timed educational messages (ITEMs, see [33]) or adapting the site automatically to address any number of demographic characteristics including language, gender, age, educational level, religious preferences, and so on [34]. These evidence-based Internet interventions require funding for development and for testing in outcome studies. Once this is done, they require funding for hosting, maintenance, and updating of the site, in addition to staff to respond to technical support questions. Thus, sustainability requires relatively modest ongoing support. There is no upper limit to how many people can use these interventions. They can be used worldwide even where no other health care services are available. The field of health care should endeavor to provide this type of intervention for the most burdensome global health problems as a minimal service available to anyone in the world at no charge to them. Localities with more resources could build upon this basic level by providing additional services from any of the following 4 levels.

#### Level 2: Guided Internet Interventions 

These evidence-based Internet interventions add live support (staff-generated emails, text messages, or phone calls; advice on which elements of the site to use; encouragement, and so on). Such interventions tend to have larger effect sizes [35]. They require staff time to prepare individualized emails or phone calls, which brings them into the “consumable” arena and sets upper limits to the number of people served. Most such sites also limit the geographical reach of the site based on who is paying for the staff providing individual guidance (local, regional, or national public health entities). But they can be stand-alone services and do not rely on having an existing health care system in place.

#### Level 3: Internet Interventions as Adjuncts to Existing Health Care

These evidence-based Internet interventions expand available interventions in existing health care settings, helping provide services to patients on long waiting lists, supporting active treatment regimens (for example, helping patients to adhere to treatment between medical appointments), and helping reduce relapse and recurrence after acute treatment ends by reminding patients to monitor their status and continue self-care.

#### Level 4: Internet Interventions to Expand Health Care Beyond Current Offerings

An example of expanding health care beyond current offerings is a health care system that would use Internet interventions to move beyond treatment into both prevention and health promotion or to provide services to patients receiving inadequate care because they do not speak the languages available from local providers.

#### Level 5: Proactive Internet Interventions 

Most current health care systems wait for people to seek help before providing it. The fifth level of intervention involves proactive identification of individuals in the community who are not already coming for services but who need interventions to prevent or manage health conditions. Internet interventions at this level would provide behavioral methods for individuals to use in the community, and, if these methods did not work, the individuals would be encouraged to seek health care at existing facilities. This level provides active case finding and preventive or treatment services in people’s homes. Such services would be considered prohibitively expensive by most health care systems, which is why they are not generally available. An experimental example using face-to-face approaches is the Outcomes of Depression International Network (ODIN) project in Europe [36,37]).

To envision how Internet interventions could contribute to the reduction of disparities in access to health care worldwide, imagine the systematic development and testing of Level 1 (automated self-help) Internet interventions for the health problems that produce the largest burden of disease in the world [38]. As these evidence-based Internet interventions are made available at no charge worldwide, individuals and public sector health care settings anywhere in the world would immediately benefit from them. This alone would contribute to the global reduction of health disparities. Settings with the economic resources and political will could build upon the Level 1 (automated self-help) evidence-based Internet interventions, augmenting care to Levels 2 through 5.

### Think Globally, Act Locally...and Share Globally 

The often-quoted dictum to “think globally” (that is, consider issues that affect humanity) and “act locally” (begin addressing these issues where you live) can be extended by using the Internet to “share globally.” For example, smoking is the number one cause of preventable death worldwide, with over five million people dying each year from tobacco-related diseases [39]. Depression is the number one cause of disability worldwide by a wide margin [38]. It is imperative that health care systems address both of these sources of unnecessary human suffering. Our research group at the University of California, San Francisco, at San Francisco General Hospital (one of our teaching hospitals) has been doing work to address these health problems with local populations for years [1]. Many individuals in San Francisco have benefited from these efforts. But once we began developing Internet interventions and testing them in randomized controlled trials, we were able to share our knowledge with hundreds of thousands of people from over 200 countries. Our outcome studies open to the entire world are difficult to categorize: are they efficacy studies or effectiveness studies? Although some of our studies are strictly randomized controlled trials, they are open to any adult in the world who wants to quit smoking. Thus, the studies are tests of effectiveness in the real world. Outcome studies in specific communities or health settings would help determine whether our findings generalize to specific locations and subpopulations. An additional benefit of Internet interventions is that they can reduce the estimated average interval between the time a health intervention is found to be effective in a research context and the time it is actually used routinely from 17 years [40] to a few hours. On the day we stopped recruitment for a recent randomized controlled smoking cessation trial, we switched our site to a participant preference trial in which any adult smoker from anywhere in the world was provided access to all active elements that had been tested in the randomized trial. Thus, the automated self-help site [41] was left up and running without charging users, while still obtaining outcome data on an ongoing fashion.

### Conclusion

To reduce health disparities worldwide, the international community should develop a system to provide evidence-based Internet interventions at no cost to the users. To launch this process, funding agencies and globally minded foundations or corporations would provide ongoing support to host and maintain automated self-help Internet interventions. The number of people who could benefit from such evidence-based Internet interventions would be massive. The return on investment on Internet interventions that can be used again and again is much higher than from provision of consumable interventions whose therapeutic power is spent after one use. The geographical reach of evidence-based Internet interventions is literally worldwide. This initiative is a worthy and feasible challenge for the 21^st^ century.

## Multimedia Appendix 1

Original PowerPoint presentation from Amsterdam conference on which the manuscript is based

## Abbreviations

ITEM: individually timed educational messages
IOM: Institute of Medicine
LMHRP: Latino Mental Health Research Program
ODIN: Outcomes of Depression International Network
SFGH: San Francisco General Hospital
UCSF: University of California, San Francisco

## References

[1] Muñoz RF, Mendelson T (2005). Toward evidence-based interventions for diverse populations: The San Francisco General Hospital prevention and treatment manuals. J Consult Clin Psychol.

[2] Muñoz RF, Lenert LL, Delucchi K, Stoddard J, Perez JE, Penilla C, Pérez-Stable EJ (2006). Toward evidence-based Internet interventions: A Spanish/English Web site for international smoking cessation trials. Nicotine Tob Res.

[3] (2010). World Health Organization.

[4] (2010). World Health Organization.

[5] (2010). World Health Organization.

[6] DiMasi JA, Hansen RW, Grabowski HG (2003). The price of innovation: new estimates of drug development costs. J Health Econ.

[7] Christensen A, Miller WR, Muñoz RF (1978). Paraprofessionals, partners, peers, paraphernalia, and print: Expanding mental health service delivery. Prof Psychol.

[8] Mrazek PB, Haggerty RJ (1994). Reducing Risks for Mental Disorders: Frontiers for Preventive Intervention Research.

[9] National Research Council and Institute of Medicine (2009). Preventing Mental, Emotional, and Behavioral Disorders Among Young People: Progress and Possibilities.

[10] Muñoz RF, Cuijpers P, Smit F, Barrera AZ, Leykin Y (2010). Prevention of major depression. Annu Rev Clin Psychol.

[11] Marks IM, Cavanagh K, Gega L (2007). Hands-On Help: Computer-Aided Psychotherapy.

[12] Marks I, Cavanagh K (2009). Computer-aided psychological treatments: evolving issues. Annu Rev Clin Psychol.

[13] Barak A, Hen L, Boniel-Nissim M, Shapira N (2008). A comprehensive review and a meta-analysis of the effectiveness of Internet-based psychotherapeutic interventions. J Technol Hum Serv.

[14] Wantland DJ, Portillo CJ, Holzemer WL, Slaughter R, McGhee EM (2004). The effectiveness of Web-based vs. non-Web-based interventions: a meta-analysis of behavioral change outcomes. J Med Internet Res.

[15] Riper H, Kramer J, Keuken M, Smit F, Schippers G, Cuijpers P (2008). Predicting successful treatment outcome of web-based self-help for problem drinkers: secondary analysis from a randomized controlled trial. J Med Internet Res.

[16] Ritterband LM, Thorndike FP, Gonder-Frederick LA, Magee JC, Bailey ET, Saylor DK, Morin CM (2009). Efficacy of an Internet-based behavioral intervention for adults with insomnia. Arch Gen Psychiatry.

[17] Christensen H, Griffiths KM (2002). The prevention of depression using the Internet. Med J Aust.

[18] Christensen H, Griffiths KM, Korten AE, Brittliffe K, Groves C (2004). A comparison of changes in anxiety and depression symptoms of spontaneous users and trial participants of a cognitive behavior therapy website. J Med Internet Res.

[19] Barak A, Klein B, Proudfoot JG (2009). Defining internet-supported therapeutic interventions. Ann Behav Med.

[20] Danaher BG, Seeley JR (2009). Methodological issues in research on web-based behavioral interventions. Ann Behav Med.

[21] Glasgow RE (2009). Enhancing the scientific foundation of internet intervention research. Ann Behav Med.

[22] Ritterband LM, Tate DF (2009). The science of internet interventions. Introduction. Ann Behav Med.

[23] Ritterband LM, Thorndike FP, Cox DJ, Kovatchev BP, Gonder-Frederick LA (2009). A behavior change model for internet interventions. Ann Behav Med.

[24] Tate DF, Finkelstein EA, Khavjou O, Gustafson A (2009). Cost effectiveness of internet interventions: review and recommendations. Ann Behav Med.

[25] (2009). University of California, San Francisco/San Francisco General Hospital.

[26] Nathan PE, Stuart SP, Dolan SL (2000). Research on psychotherapy efficacy and effectiveness: between Scylla and Charybdis?. Psychol Bull.

[27] Schroeder SA (2005). What to do with a patient who smokes. JAMA.

[28] Eysenbach G (2005). The law of attrition. J Med Internet Res.

[29] Muñoz RF, Barrera AZ, Delucchi K, Penilla C, Torres LD, Pérez-Stable EJ (2009). International Spanish/English Internet smoking cessation trial yields 20% abstinence rates at 1 year. Nicotine Tob Res.

[30] Barrera AZ, Pérez-Stable EJ, Delucchi KL, Muñoz RF (2009). Global reach of an Internet smoking cessation intervention among Spanish- and English-speaking smokers from 157 countries. Int J Environ Res Public Health.

[31] (2010). Beacon.

[32] Chan-Olmsted SM, Kim Y (2002). The PBS brand versus cable brands: Assessing the brand image of public television in a multichannel environment. Journal of Broadcasting & Electronic Media.

[33] Lenert L, Muñoz RF, Perez JE, Bansod A (2004). Automated e-mail messaging as a tool for improving quit rates in an internet smoking cessation intervention. J Am Med Inform Assoc.

[34] Strecher VJ, McClure JB, Alexander GL, Chakraborty B, Nair VN, Konkel JM, Greene SM, Collins LM, Carlier CC, Wiese CJ, Little RJ, Pomerleau CS, Pomerleau OF (2008). Web-based smoking-cessation programs: results of a randomized trial. Am J Prev Med.

[35] Cuijpers P, Donker T, van Straten A, Li J, Andersson G (2010). Is guided self-help as effective as face-to-face psychotherapy for depression and anxiety disorders? A systematic review and meta-analysis of comparative outcome studies. Psychol Med.

[36] Dowrick C, Casey P, Dalgard O, Hosman C, Lehtinen V, Vázquez-Barquero JL, Wilkinson G (1998). Outcomes of Depression International Network (ODIN). Background, methods and field trials. ODIN Group. Br J Psychiatry.

[37] Dowrick C, Dunn G, Ayuso-Mateos JL, Dalgard OS, Page H, Lehtinen V, Casey P, Wilkinson C, Vazquez-Barquero JL, Wilkinson G (2000). Problem solving treatment and group psychoeducation for depression: multicentre randomised controlled trial. Outcomes of Depression International Network (ODIN) Group. BMJ.

[38] Murray CJL, Lopez AD (1996). The global burden of disease: Summary.

[39] (2010). World Health Organization.

[40] Committee on Quality of Health Care in America, Institute of Medicine (2001). Crossing the Quality Chasm: A Hew Health System for the 21st Century.

[41] (2010). University of California, San Francisco/San Francisco General Hospital.

