# Supplementary material for: Using Evidence-Based Internet Interventions to Reduce Health Disparities Worldwide
Source: J Med Internet Res. 2010 Dec 17;12(5):e60. doi: 10.2196/jmir.1463 (PMC3057307; doi:10.2196/jmir.1463)
Supplement: Supplementary file 1 [file jmir_v12i5e60_app1.ppt]

## Slide 1
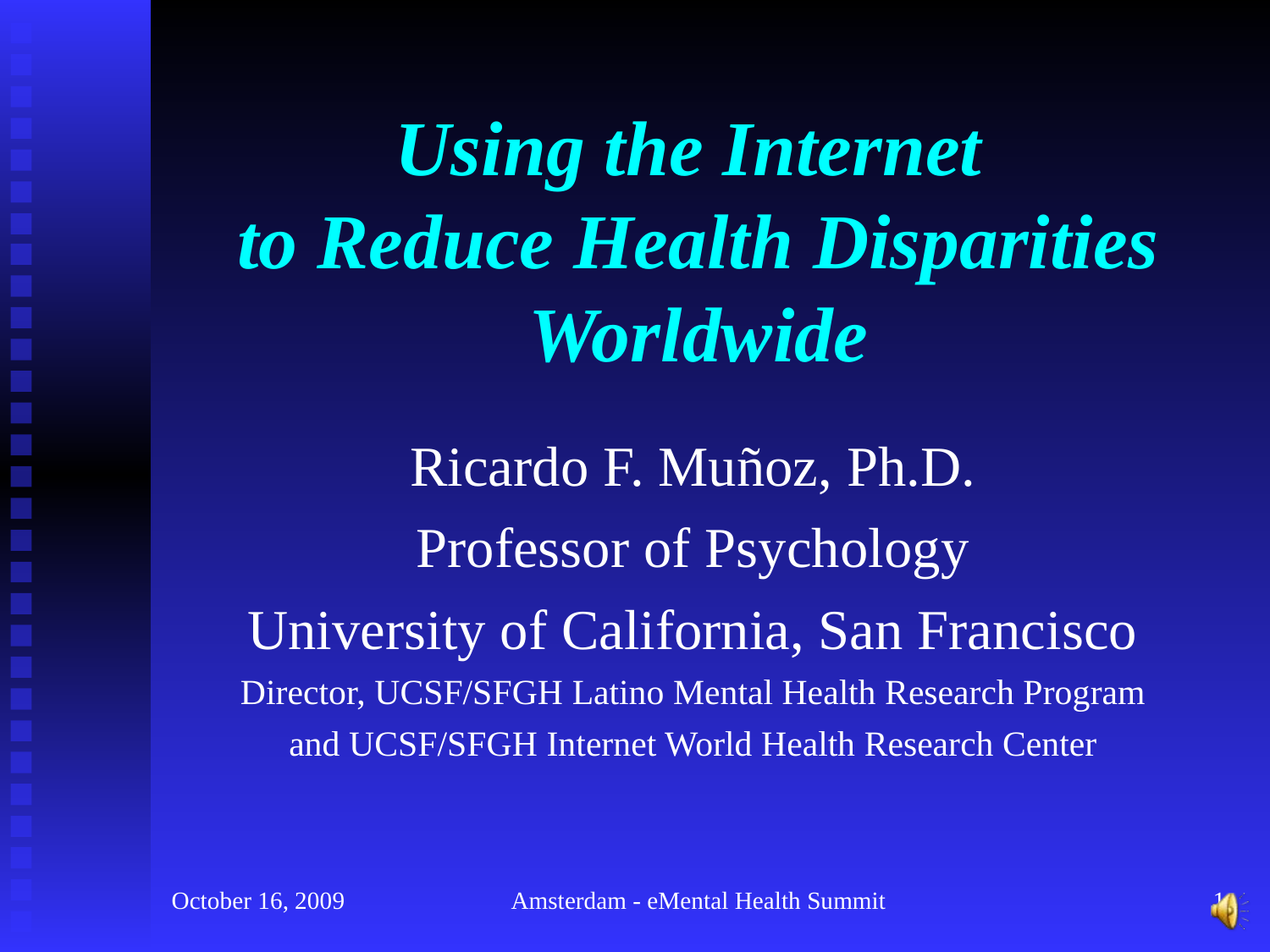

# Using the Internet to Reduce Health Disparities Worldwide
Ricardo F. Muñoz, Ph.D.
Professor of Psychology
University of California, San Francisco
Director, UCSF/SFGH Latino Mental Health Research Program
and UCSF/SFGH Internet World Health Research Center
October 16, 2009
Amsterdam - eMental Health Summit
1

## Slide 2
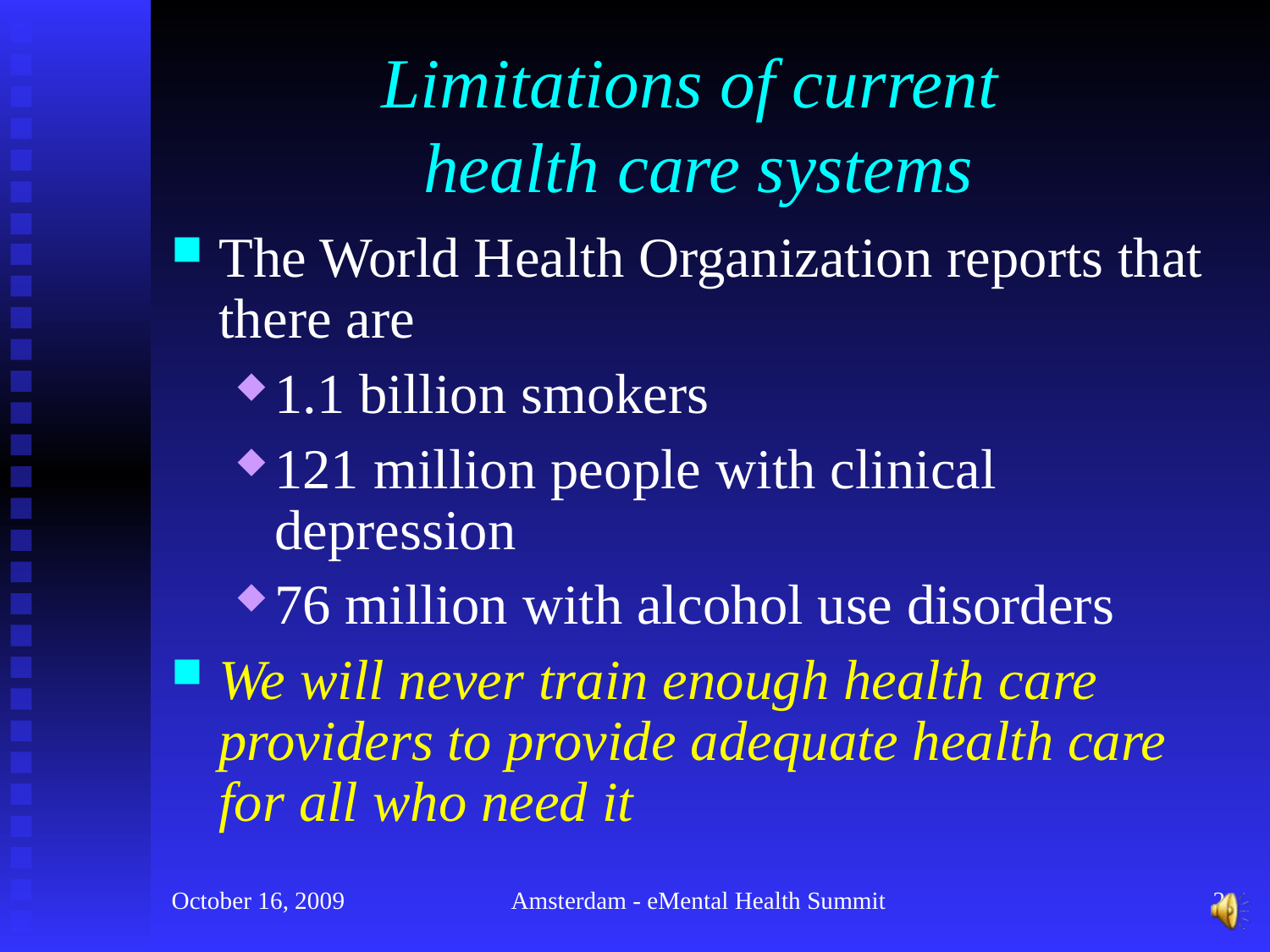

# Limitations of current health care systems
The World Health Organization reports that there are
1.1 billion smokers
121 million people with clinical depression
76 million with alcohol use disorders
We will never train enough health care providers to provide adequate health care for all who need it
October 16, 2009
Amsterdam - eMental Health Summit
2

## Slide 3
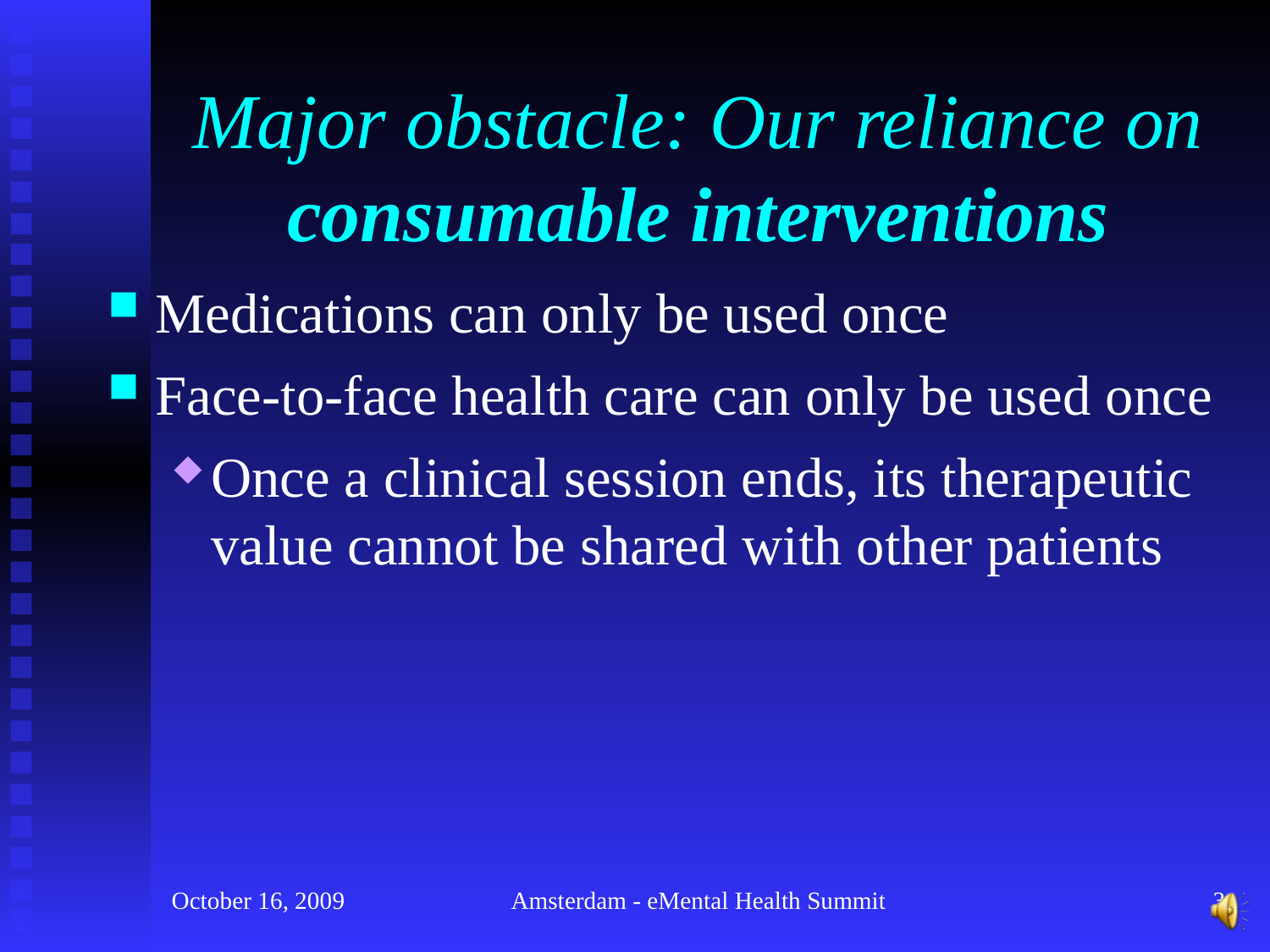

# Major obstacle: Our reliance on consumable interventions
Medications can only be used once
Face-to-face health care can only be used once
Once a clinical session ends, its therapeutic value cannot be shared with other patients
October 16, 2009
Amsterdam - eMental Health Summit
3

## Slide 4
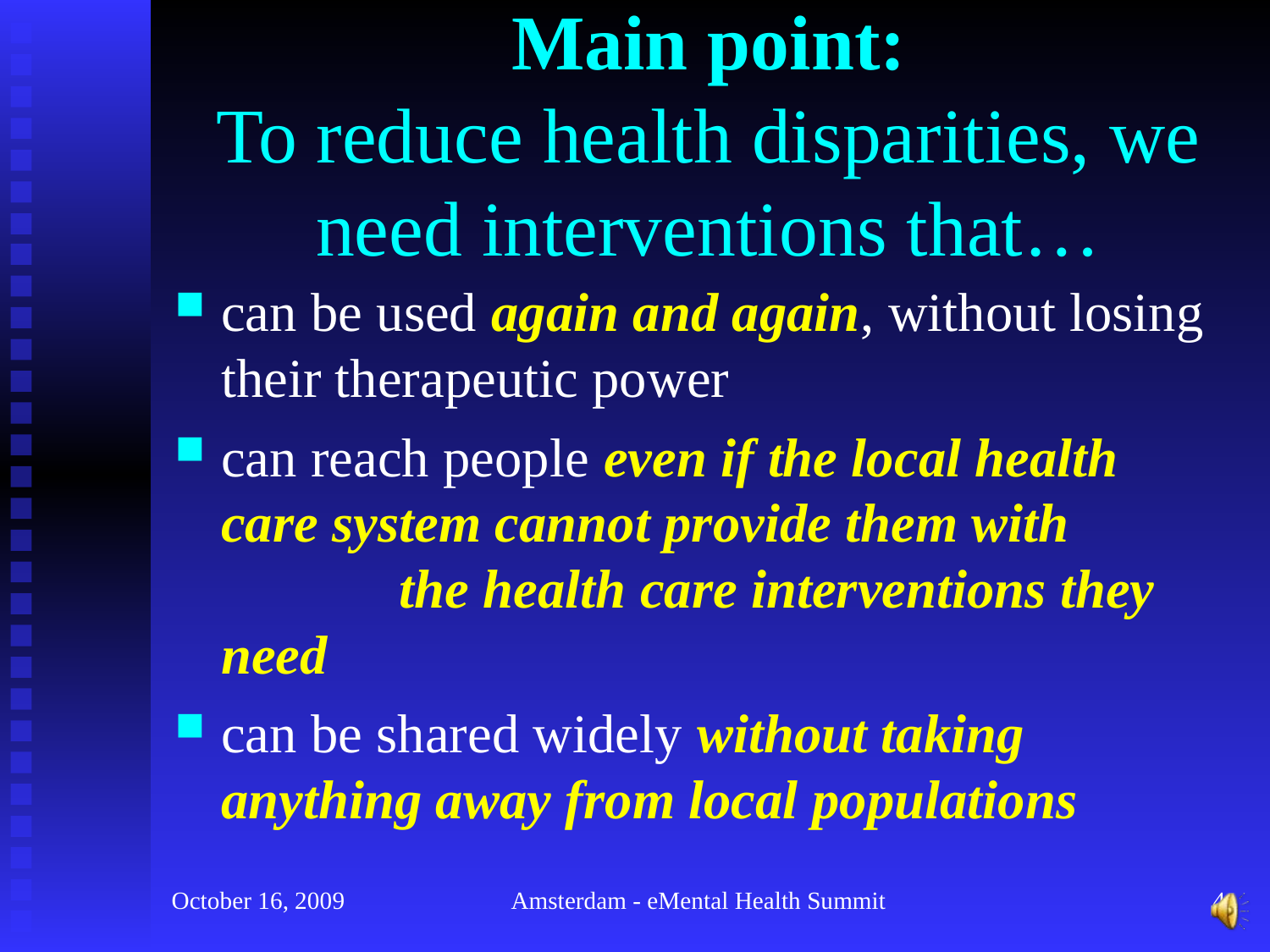

# Main point: To reduce health disparities, we need interventions that…
can be used again and again, without losing their therapeutic power
can reach people even if the local health care system cannot provide them with the health care interventions they need
can be shared widely without taking anything away from local populations
October 16, 2009
Amsterdam - eMental Health Summit
4

## Slide 5
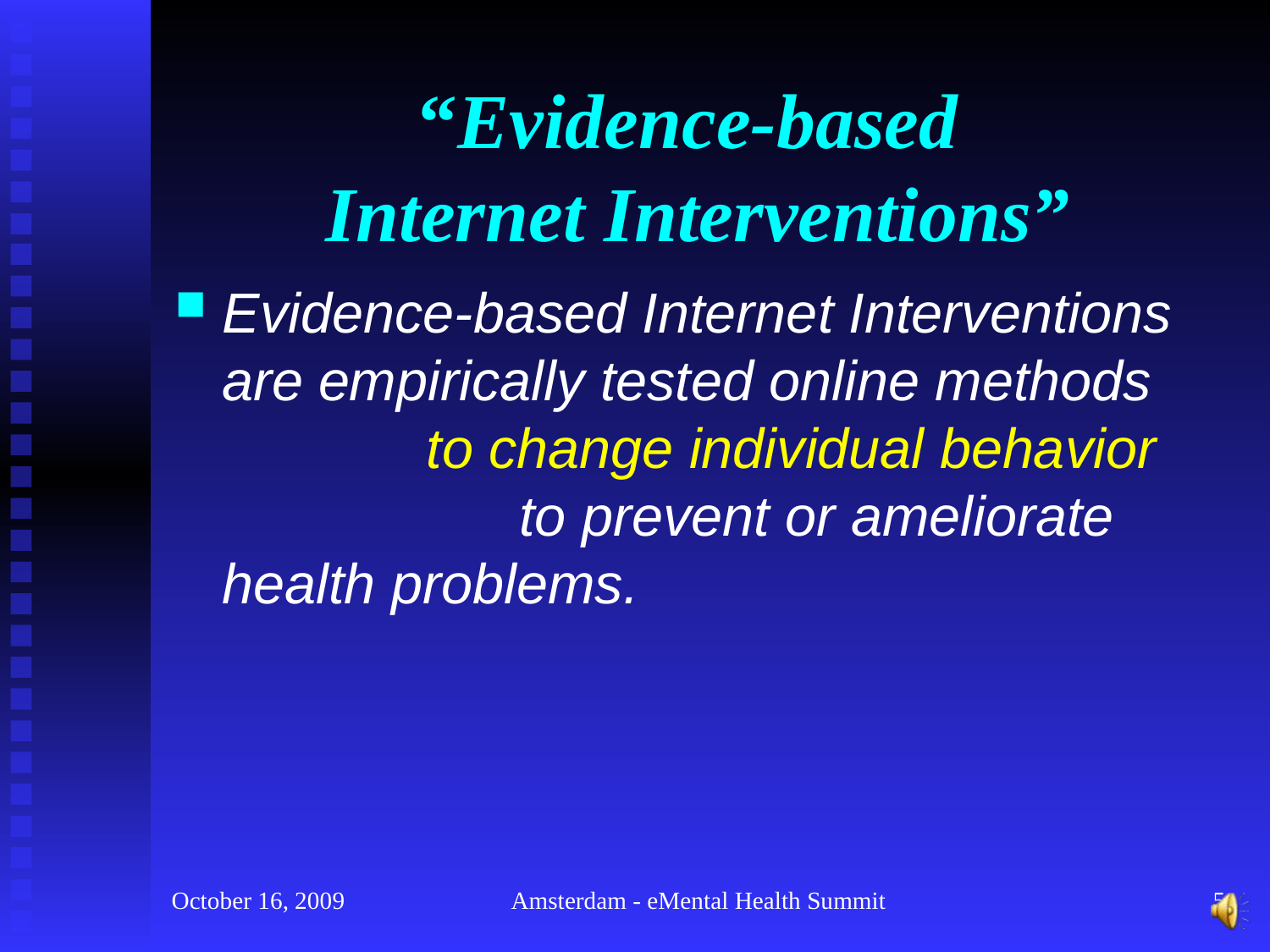

# “Evidence-based Internet Interventions”
Evidence-based Internet Interventions are empirically tested online methods to change individual behavior to prevent or ameliorate health problems.
October 16, 2009
Amsterdam - eMental Health Summit
5

## Slide 6
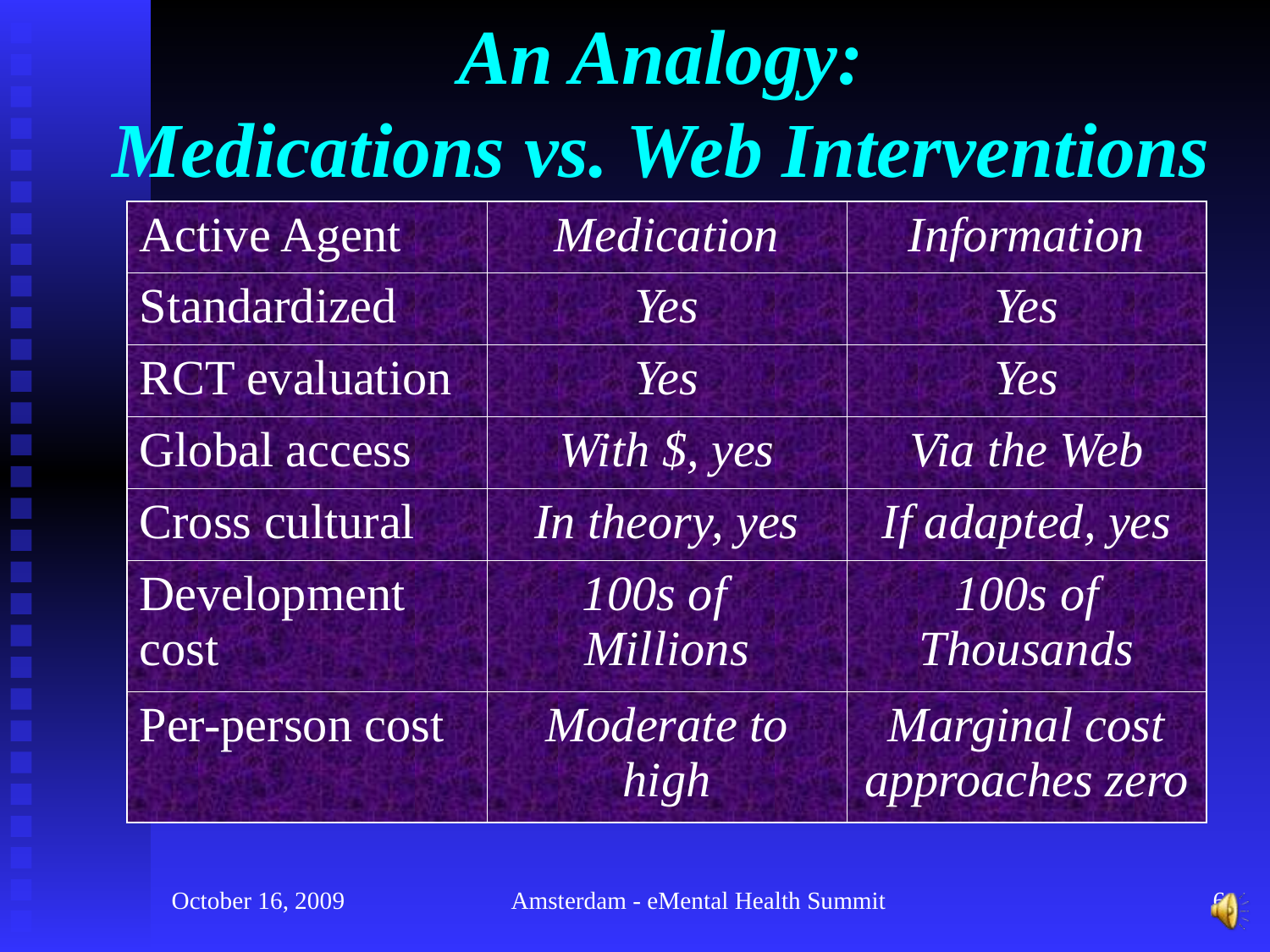

An Analogy:
Medications vs. Web Interventions
| Active Agent | Medication | Information |
| --- | --- | --- |
| Standardized | Yes | Yes |
| RCT evaluation | Yes | Yes |
| Global access | With $, yes | Via the Web |
| Cross cultural | In theory, yes | If adapted, yes |
| Development cost | 100s of Millions | 100s of Thousands |
| Per-person cost | Moderate to high | Marginal cost approaches zero |
October 16, 2009
Amsterdam - eMental Health Summit
6

## Slide 7
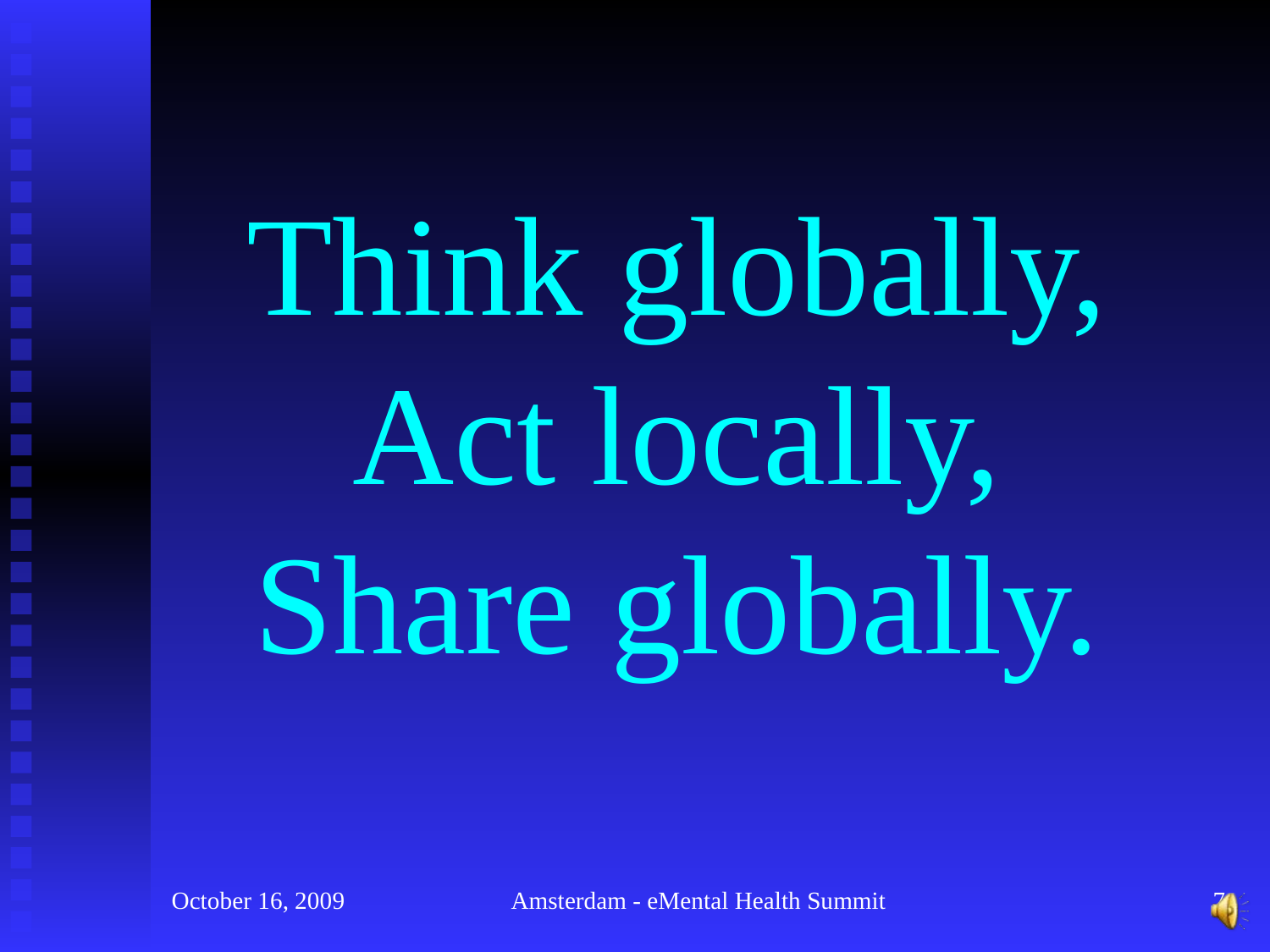

# Think globally, Act locally, Share globally.
October 16, 2009
Amsterdam - eMental Health Summit
7

## Slide 8
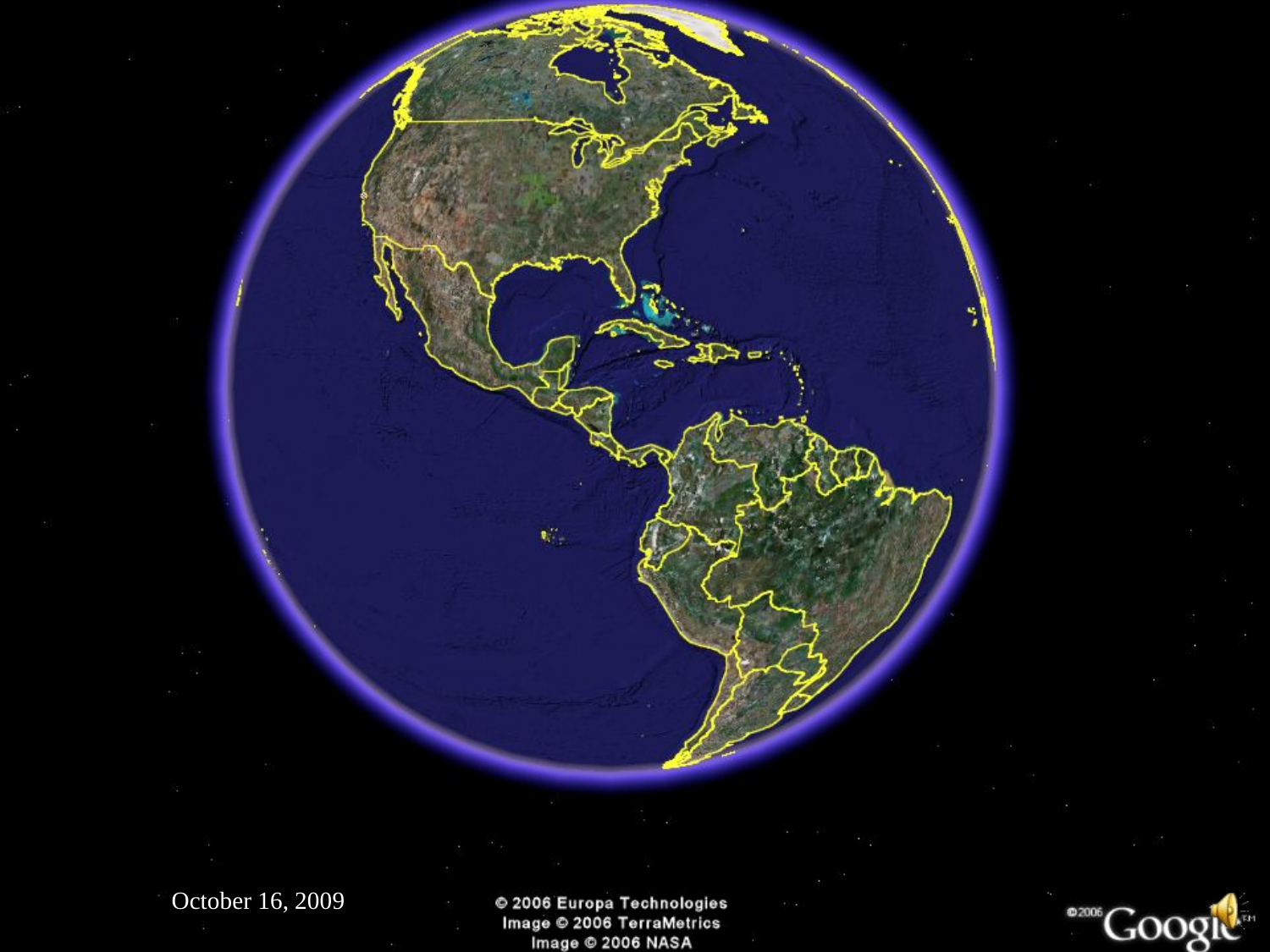

October 16, 2009
Amsterdam - eMental Health Summit
8

## Slide 9
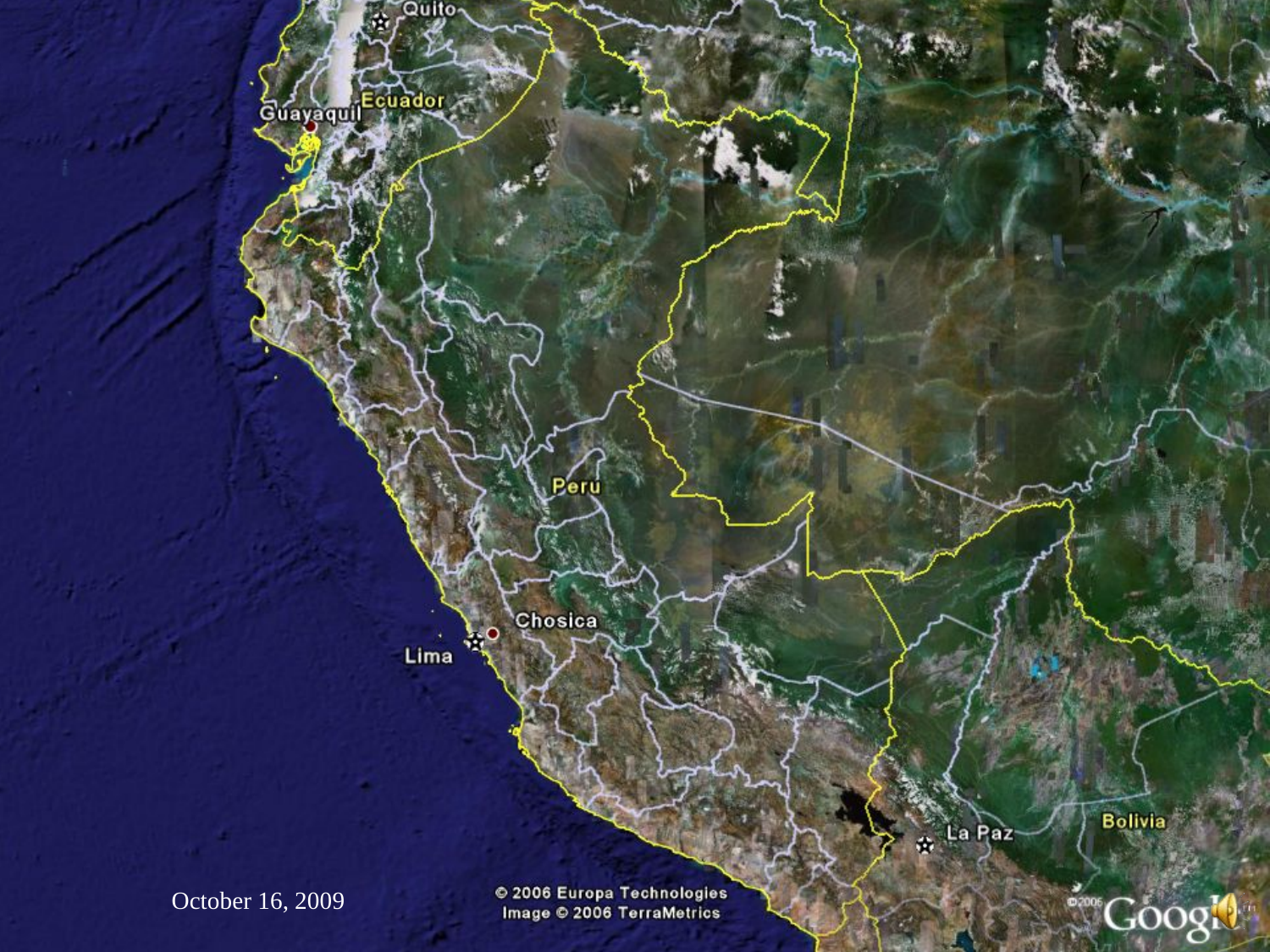

October 16, 2009
Amsterdam - eMental Health Summit
9

## Slide 10
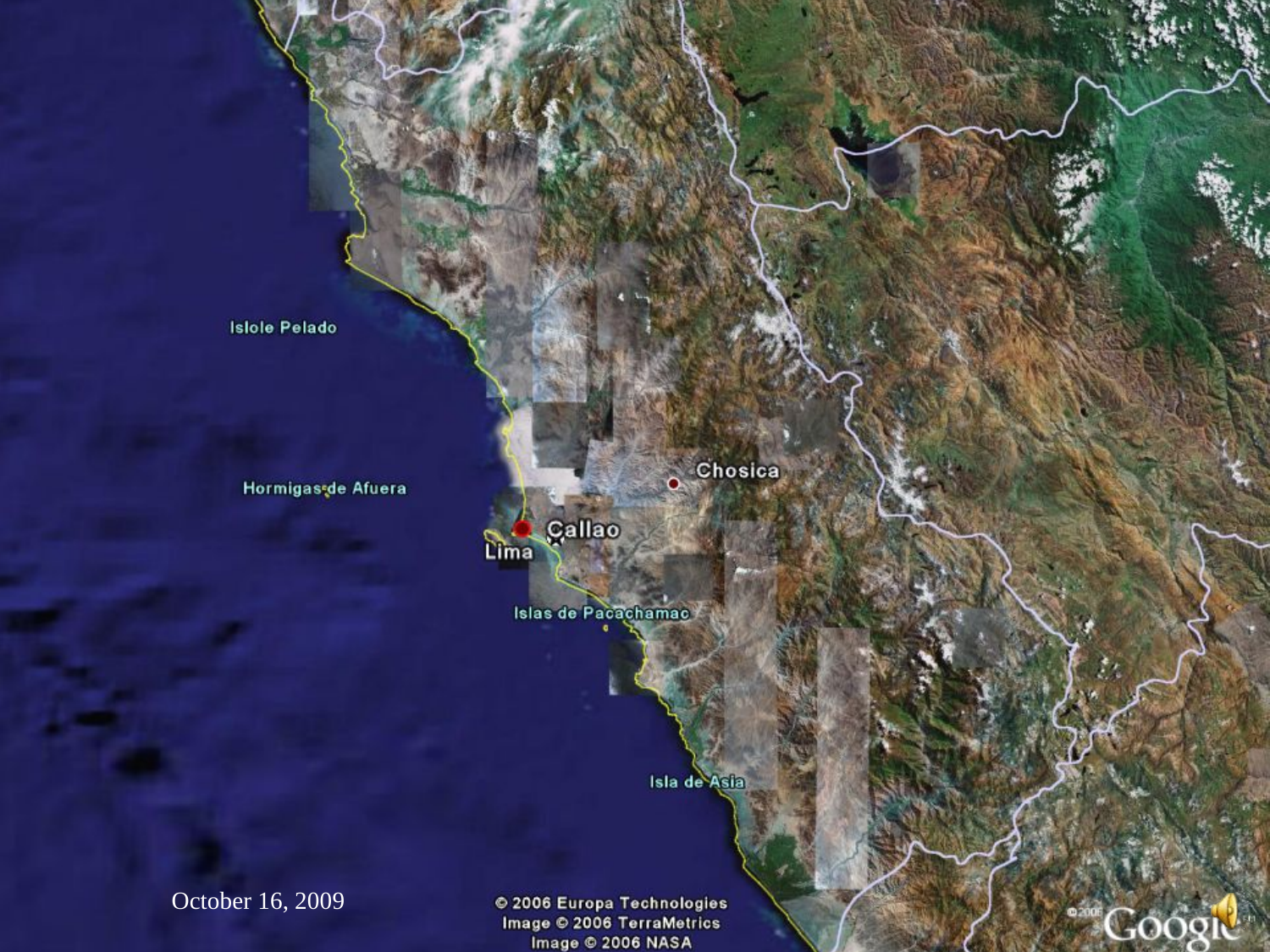

October 16, 2009
Amsterdam - eMental Health Summit
10

## Slide 11
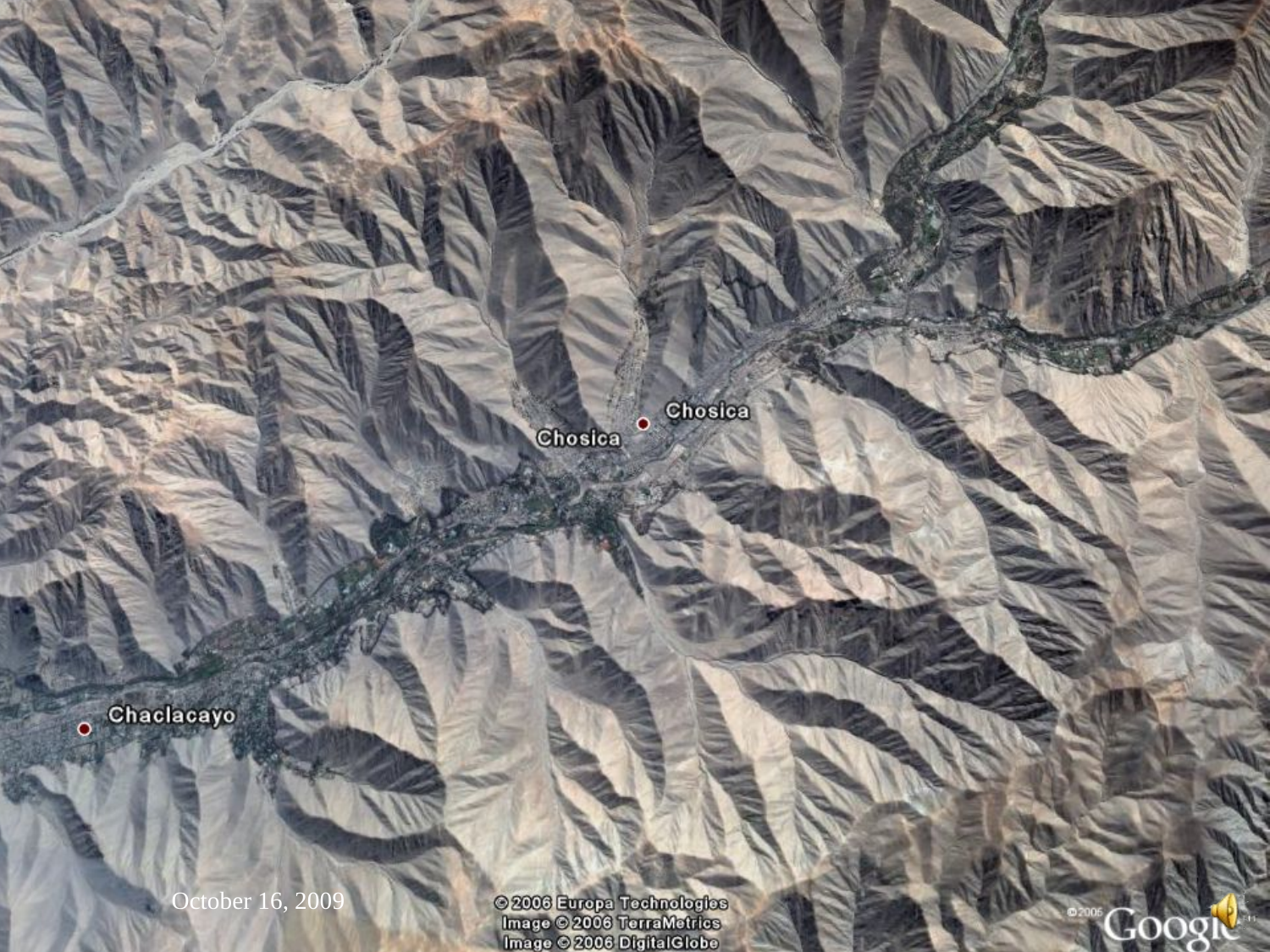

October 16, 2009
Amsterdam - eMental Health Summit
11

## Slide 12
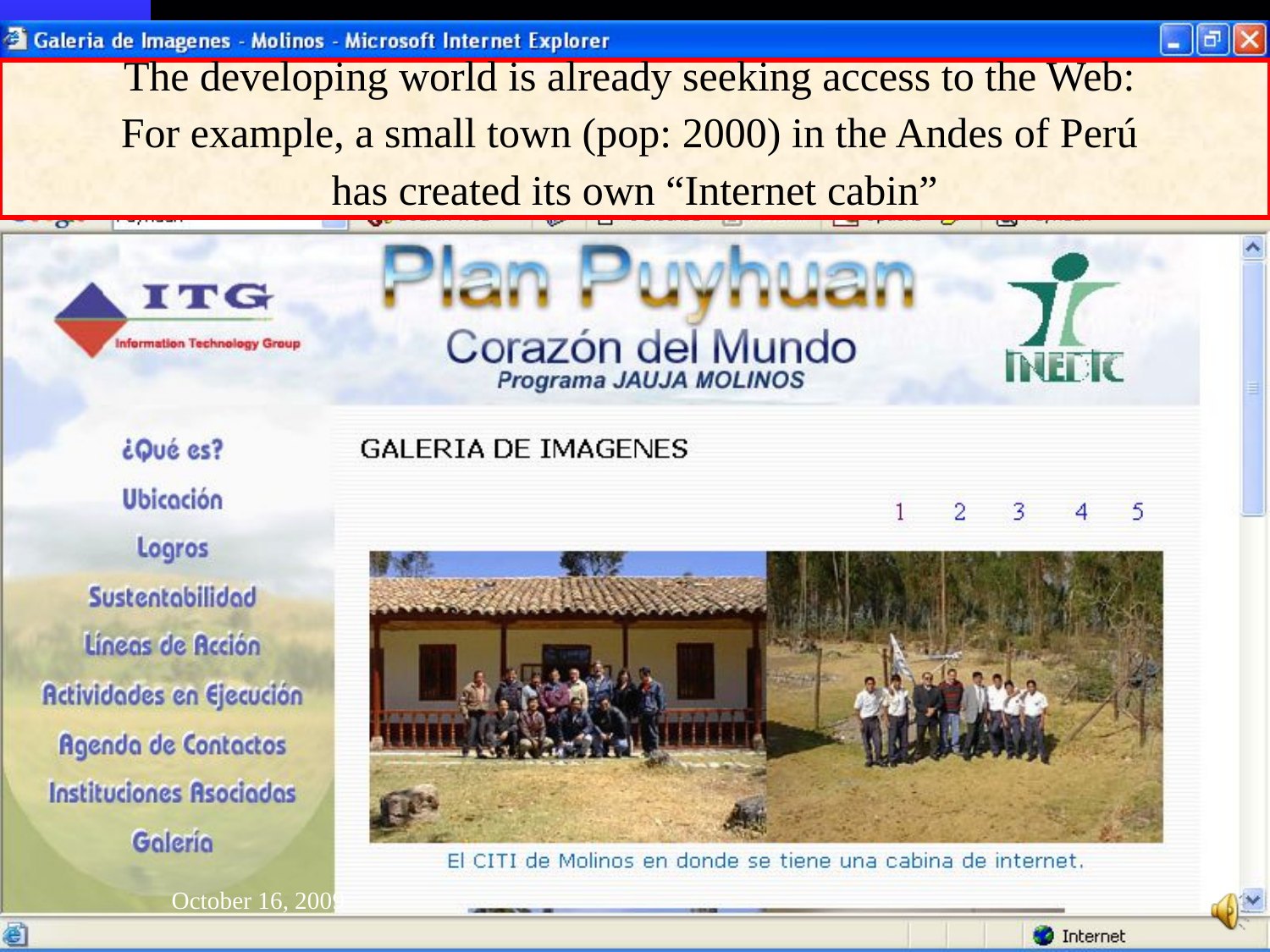

The developing world is already seeking access to the Web:
For example, a small town (pop: 2000) in the Andes of Perú
has created its own “Internet cabin”
October 16, 2009
Amsterdam - eMental Health Summit
12

## Slide 13
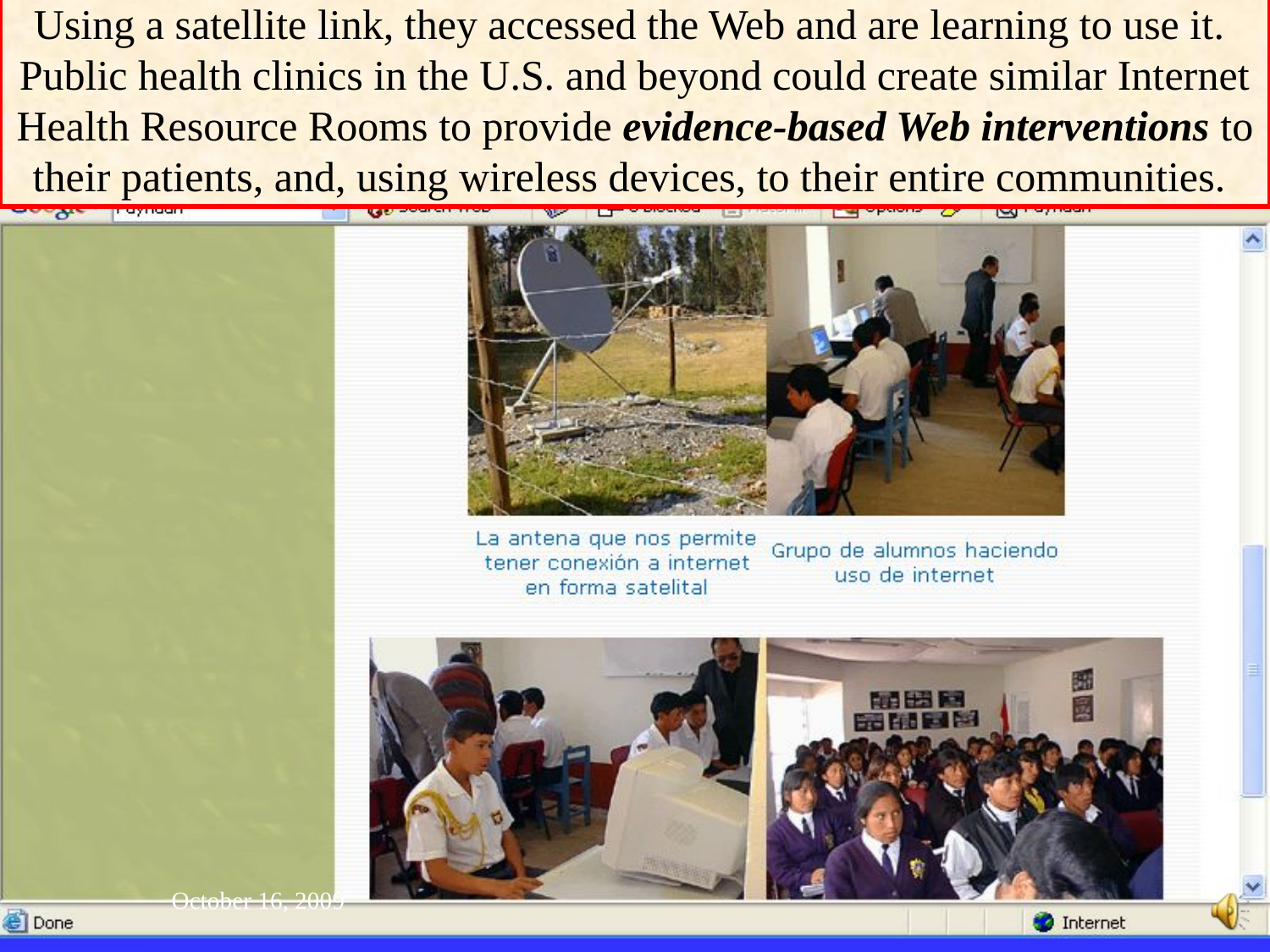

Using a satellite link, they accessed the Web and are learning to use it. Public health clinics in the U.S. and beyond could create similar Internet Health Resource Rooms to provide evidence-based Web interventions to their patients, and, using wireless devices, to their entire communities.
October 16, 2009
Amsterdam - eMental Health Summit
13

## Slide 14
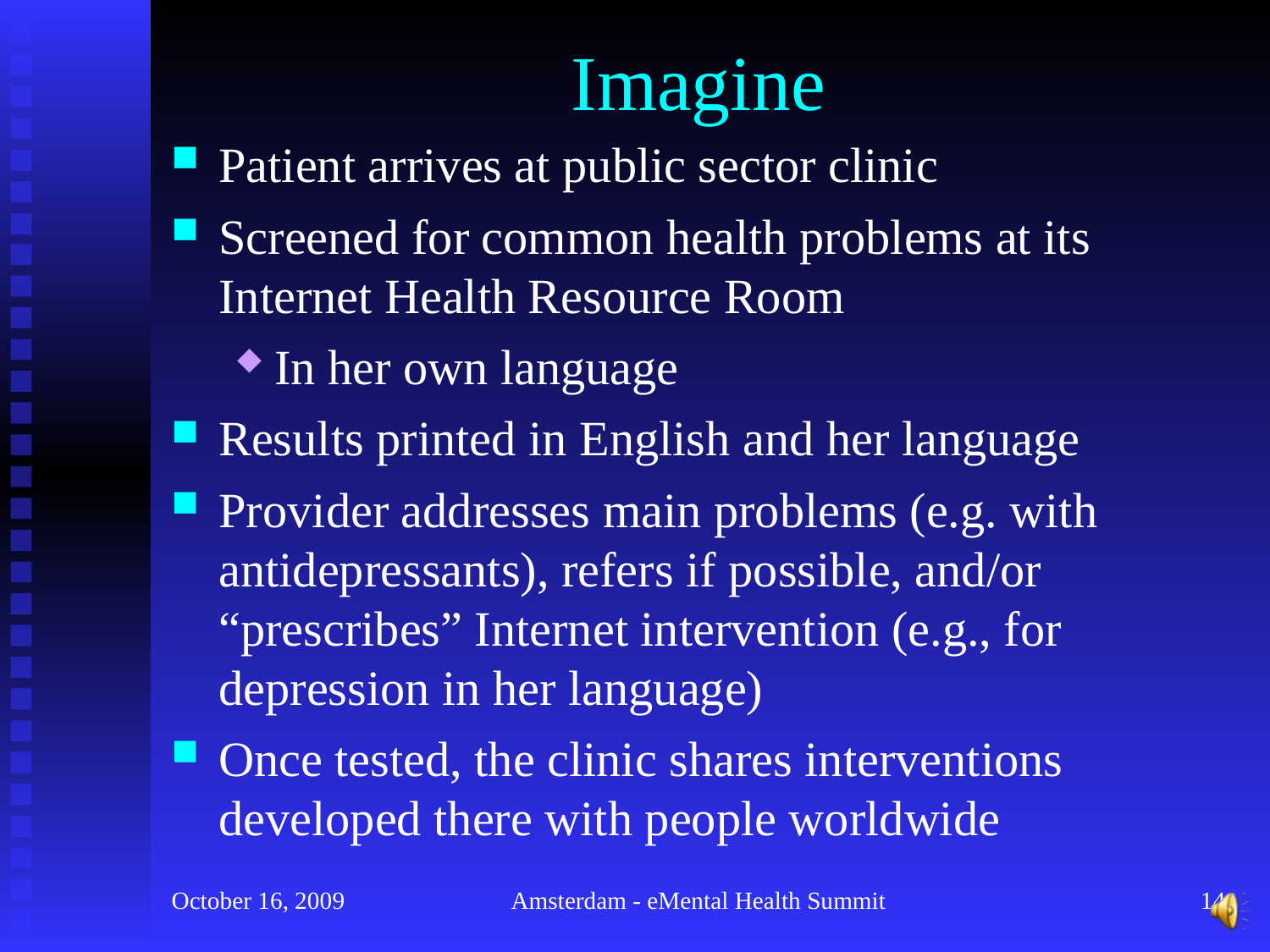

# Imagine
Patient arrives at public sector clinic
Screened for common health problems at its Internet Health Resource Room
In her own language
Results printed in English and her language
Provider addresses main problems (e.g. with antidepressants), refers if possible, and/or “prescribes” Internet intervention (e.g., for depression in her language)
Once tested, the clinic shares interventions developed there with people worldwide
October 16, 2009
Amsterdam - eMental Health Summit
14

## Slide 15
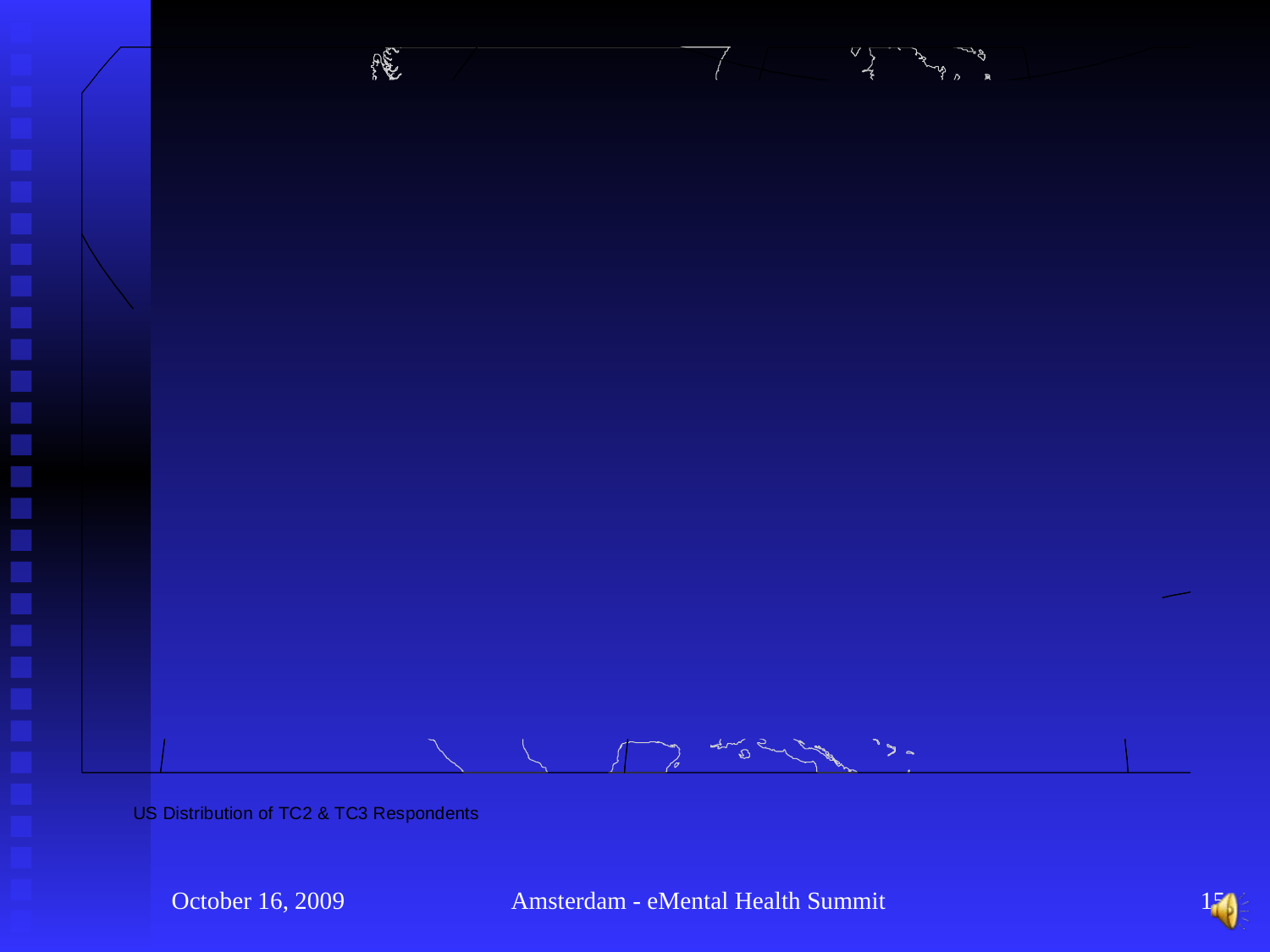

October 16, 2009
Amsterdam - eMental Health Summit
15

## Slide 16
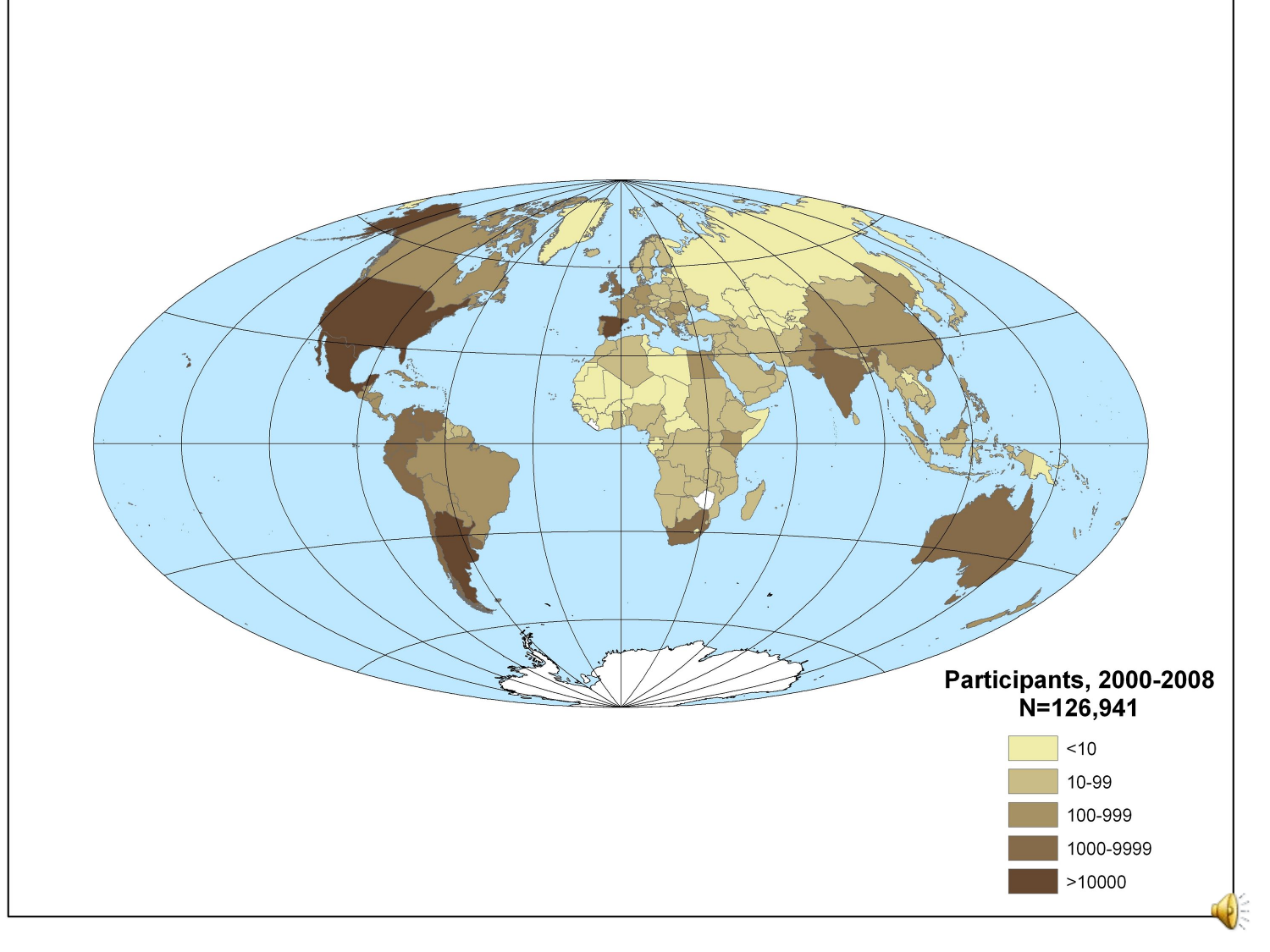

October 16, 2009
Amsterdam - eMental Health Summit
16

## Slide 17
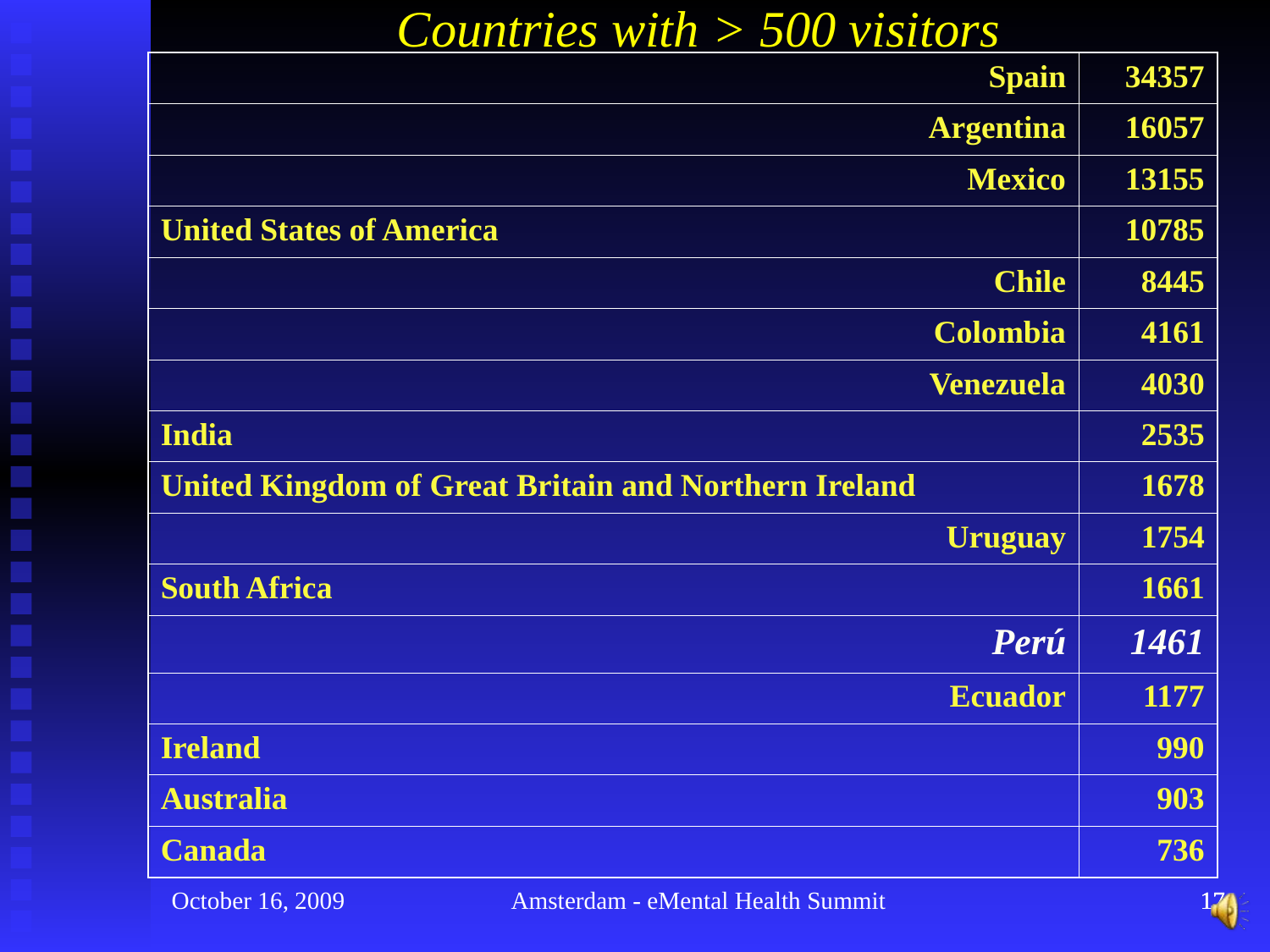

# Countries with > 500 visitors
| Spain | 34357 |
| --- | --- |
| Argentina | 16057 |
| Mexico | 13155 |
| United States of America | 10785 |
| Chile | 8445 |
| Colombia | 4161 |
| Venezuela | 4030 |
| India | 2535 |
| United Kingdom of Great Britain and Northern Ireland | 1678 |
| Uruguay | 1754 |
| South Africa | 1661 |
| Perú | 1461 |
| Ecuador | 1177 |
| Ireland | 990 |
| Australia | 903 |
| Canada | 736 |
October 16, 2009
Amsterdam - eMental Health Summit
<number>
<number>
17

## Slide 18
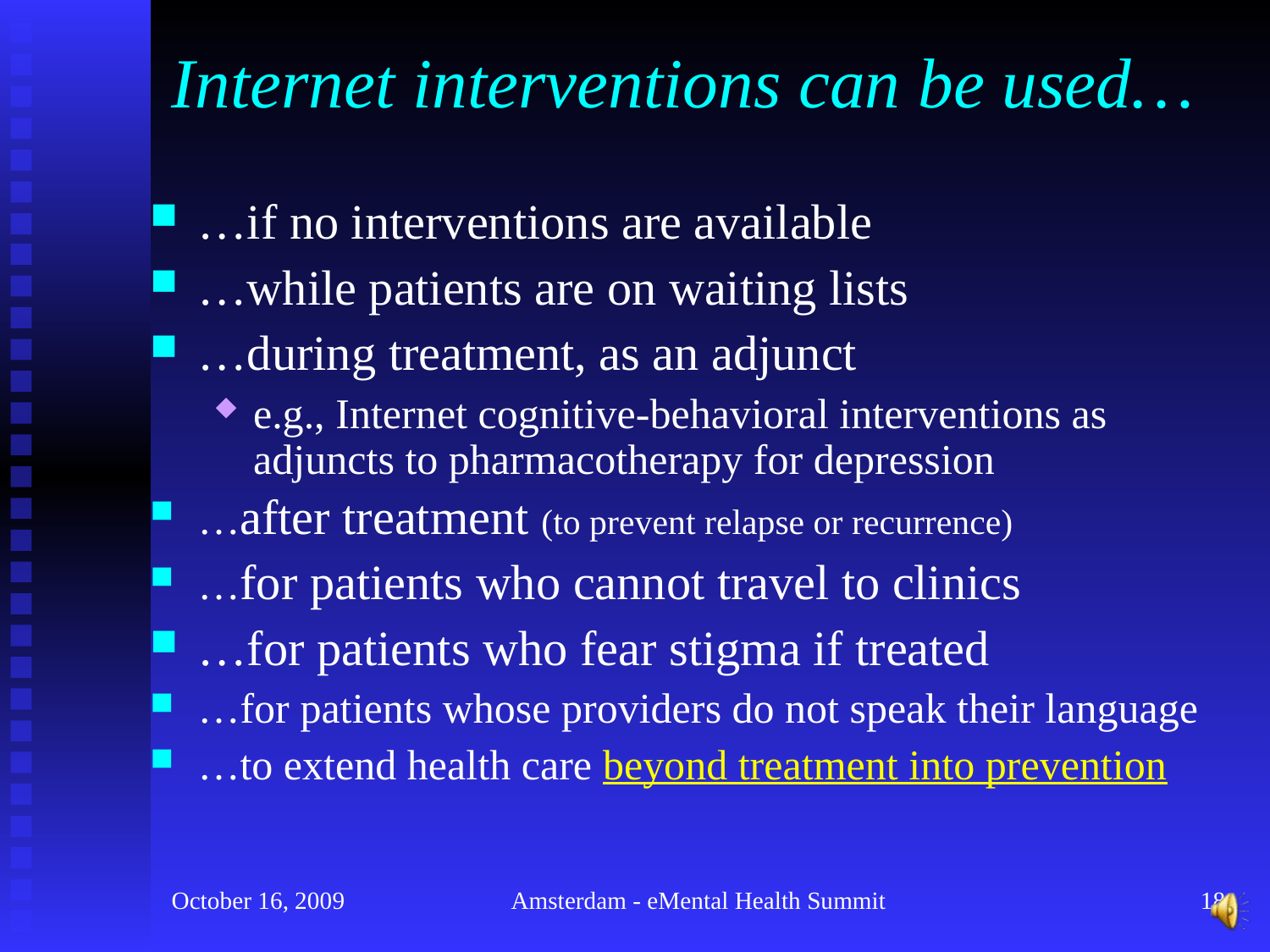

# Internet interventions can be used…
…if no interventions are available
…while patients are on waiting lists
…during treatment, as an adjunct
e.g., Internet cognitive-behavioral interventions as adjuncts to pharmacotherapy for depression
…after treatment (to prevent relapse or recurrence)
…for patients who cannot travel to clinics
…for patients who fear stigma if treated
…for patients whose providers do not speak their language
…to extend health care beyond treatment into prevention
October 16, 2009
Amsterdam - eMental Health Summit
18

## Slide 19
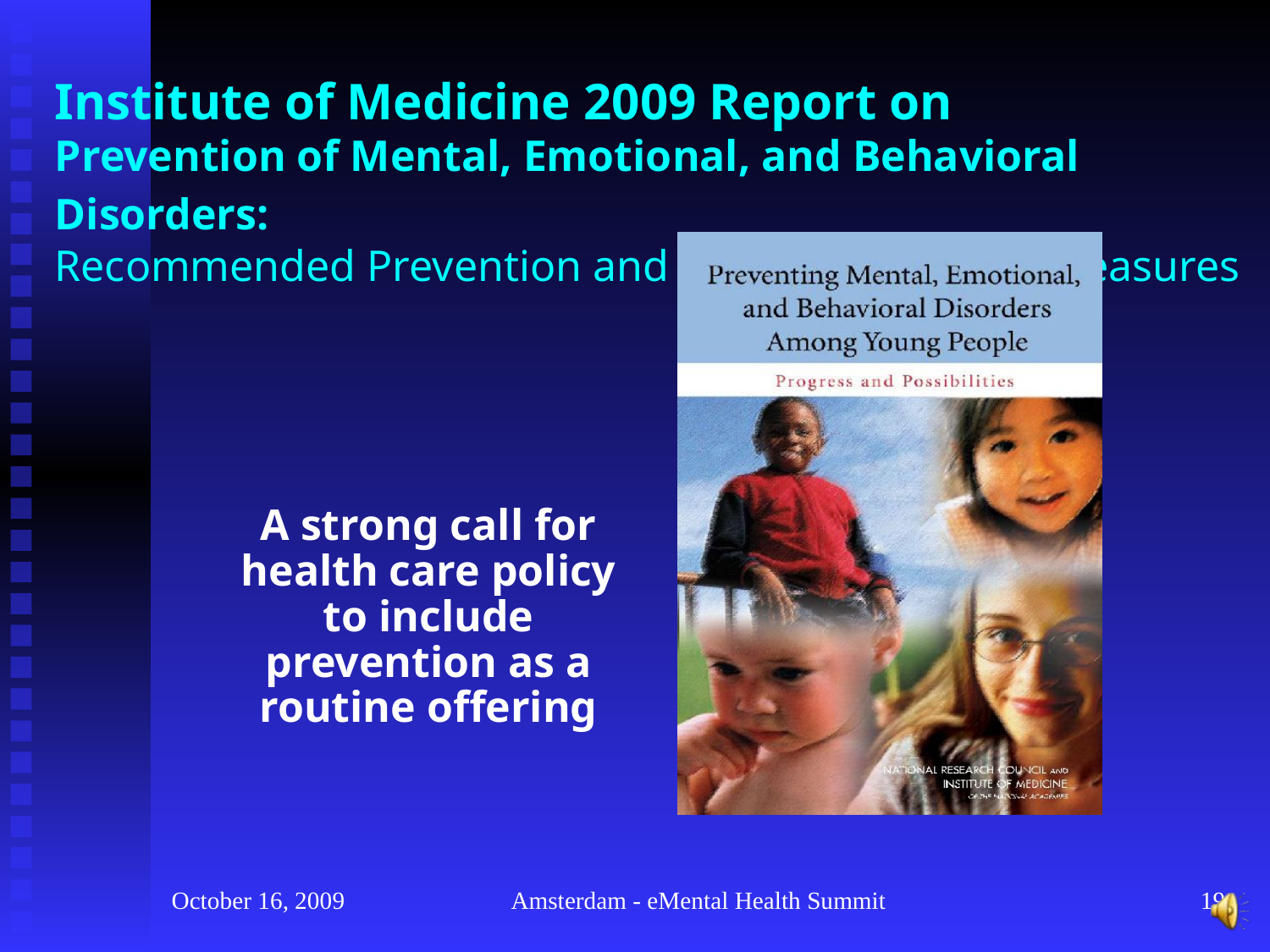

Institute of Medicine 2009 Report on
Prevention of Mental, Emotional, and Behavioral Disorders:
Recommended Prevention and Early Intervention Measures
#
A strong call for health care policy to include prevention as a routine offering
October 16, 2009
Amsterdam - eMental Health Summit
19

## Slide 20
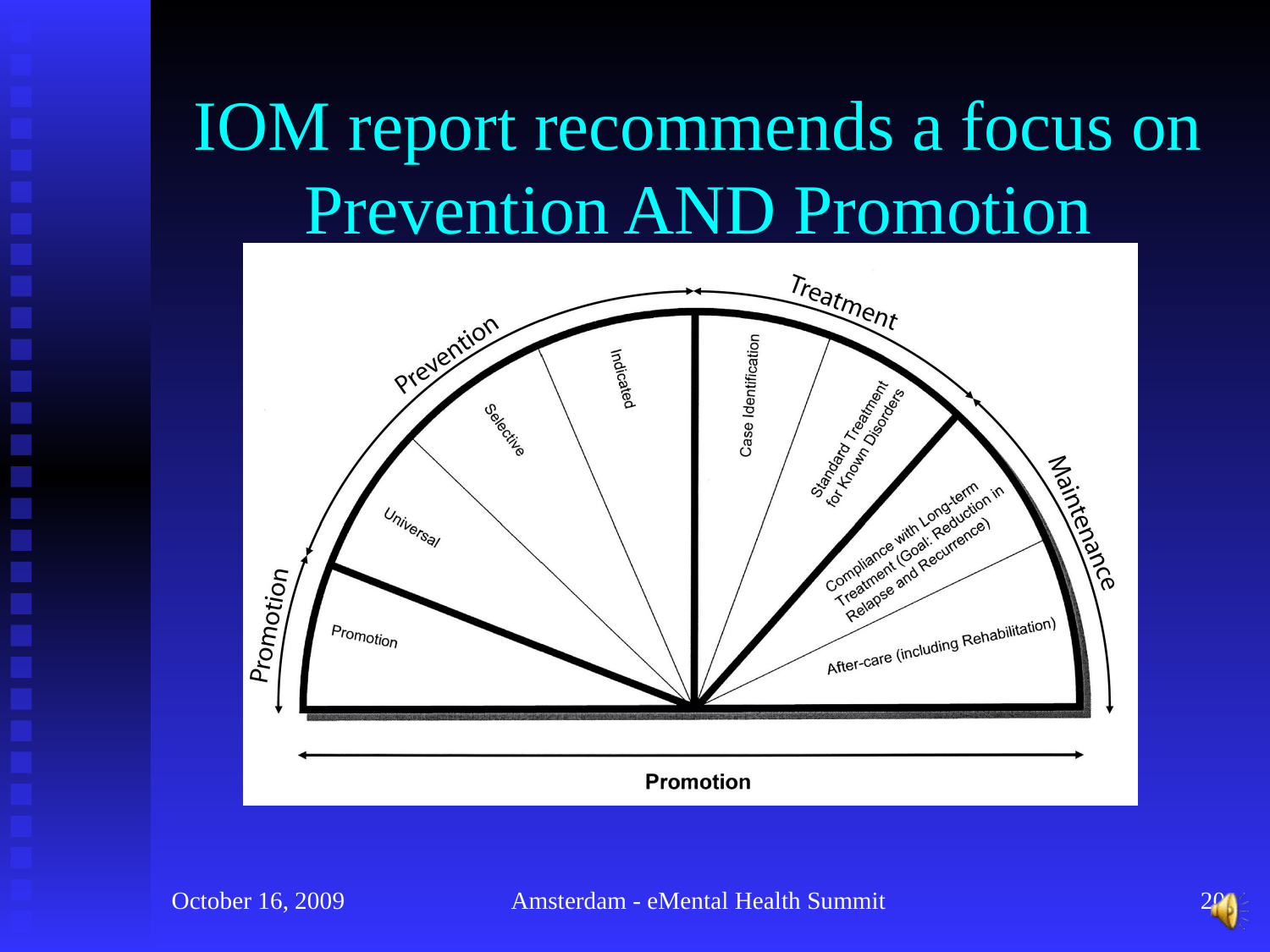

# IOM report recommends a focus on Prevention AND Promotion
October 16, 2009
Amsterdam - eMental Health Summit
20

## Slide 21
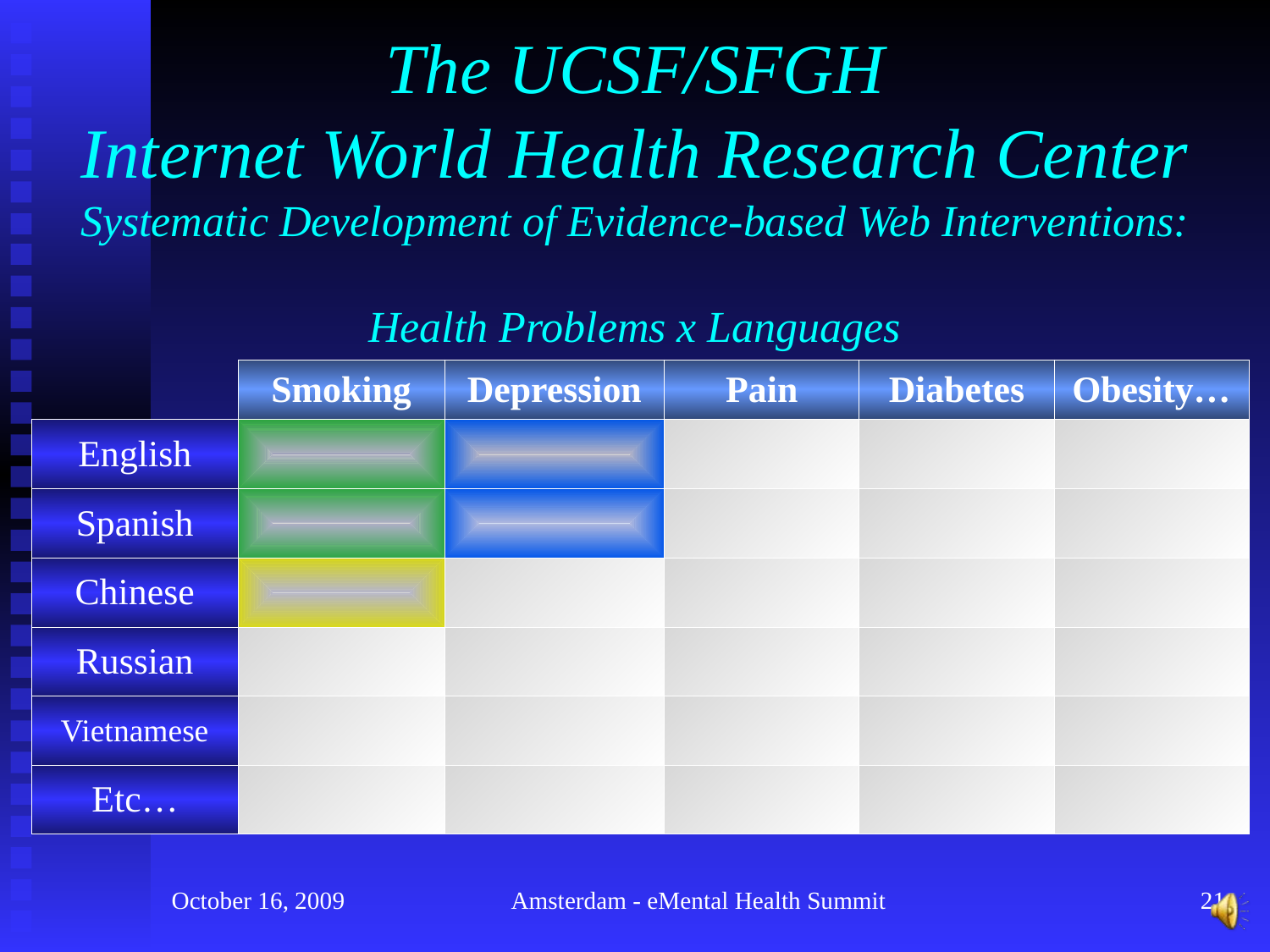

# The UCSF/SFGH Internet World Health Research Center Systematic Development of Evidence-based Web Interventions: Health Problems x Languages
| | Smoking | Depression | Pain | Diabetes | Obesity… |
| --- | --- | --- | --- | --- | --- |
| English | | | | | |
| Spanish | | | | | |
| Chinese | | | | | |
| Russian | | | | | |
| Vietnamese | | | | | |
| Etc… | | | | | |
October 16, 2009
Amsterdam - eMental Health Summit
21

## Slide 22
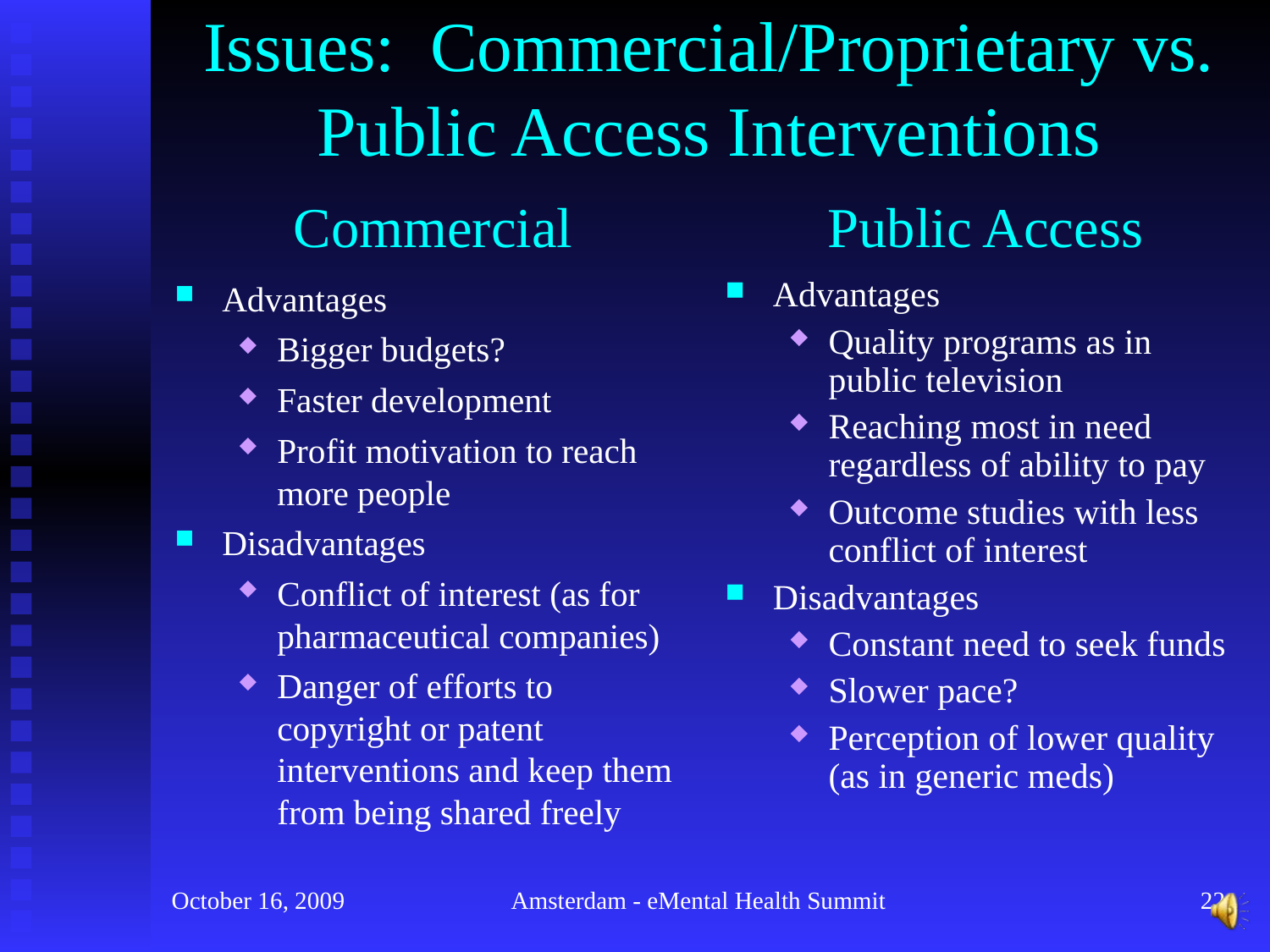

# Issues: Commercial/Proprietary vs. Public Access Interventions Commercial Public Access
Advantages
Bigger budgets?
Faster development
Profit motivation to reach more people
Disadvantages
Conflict of interest (as for pharmaceutical companies)
Danger of efforts to copyright or patent interventions and keep them from being shared freely
Advantages
Quality programs as in public television
Reaching most in need regardless of ability to pay
Outcome studies with less conflict of interest
Disadvantages
Constant need to seek funds
Slower pace?
Perception of lower quality (as in generic meds)
October 16, 2009
Amsterdam - eMental Health Summit
22

## Slide 23
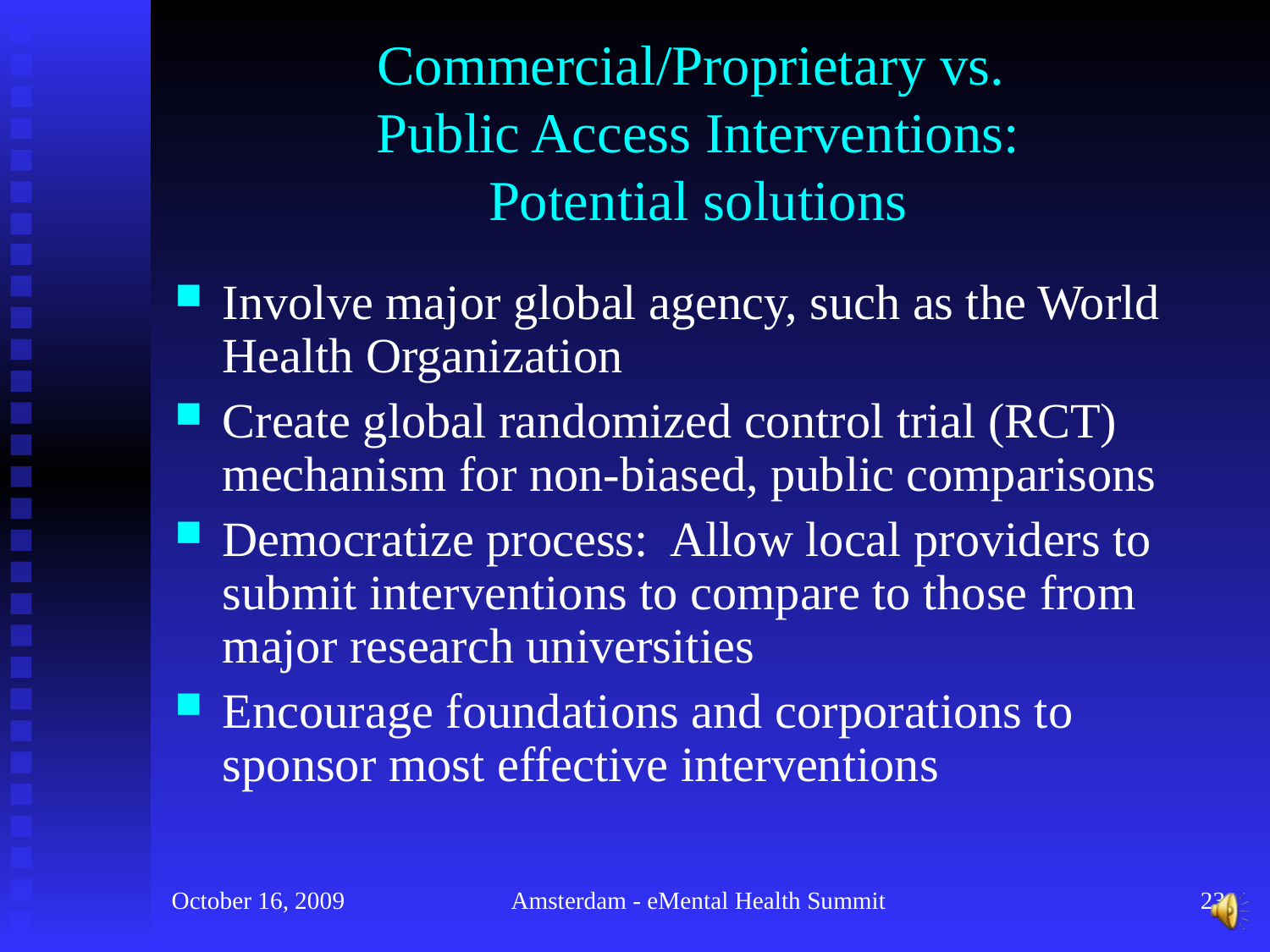

# Commercial/Proprietary vs. Public Access Interventions: Potential solutions
Involve major global agency, such as the World Health Organization
Create global randomized control trial (RCT) mechanism for non-biased, public comparisons
Democratize process: Allow local providers to submit interventions to compare to those from major research universities
Encourage foundations and corporations to sponsor most effective interventions
October 16, 2009
Amsterdam - eMental Health Summit
23

## Slide 24
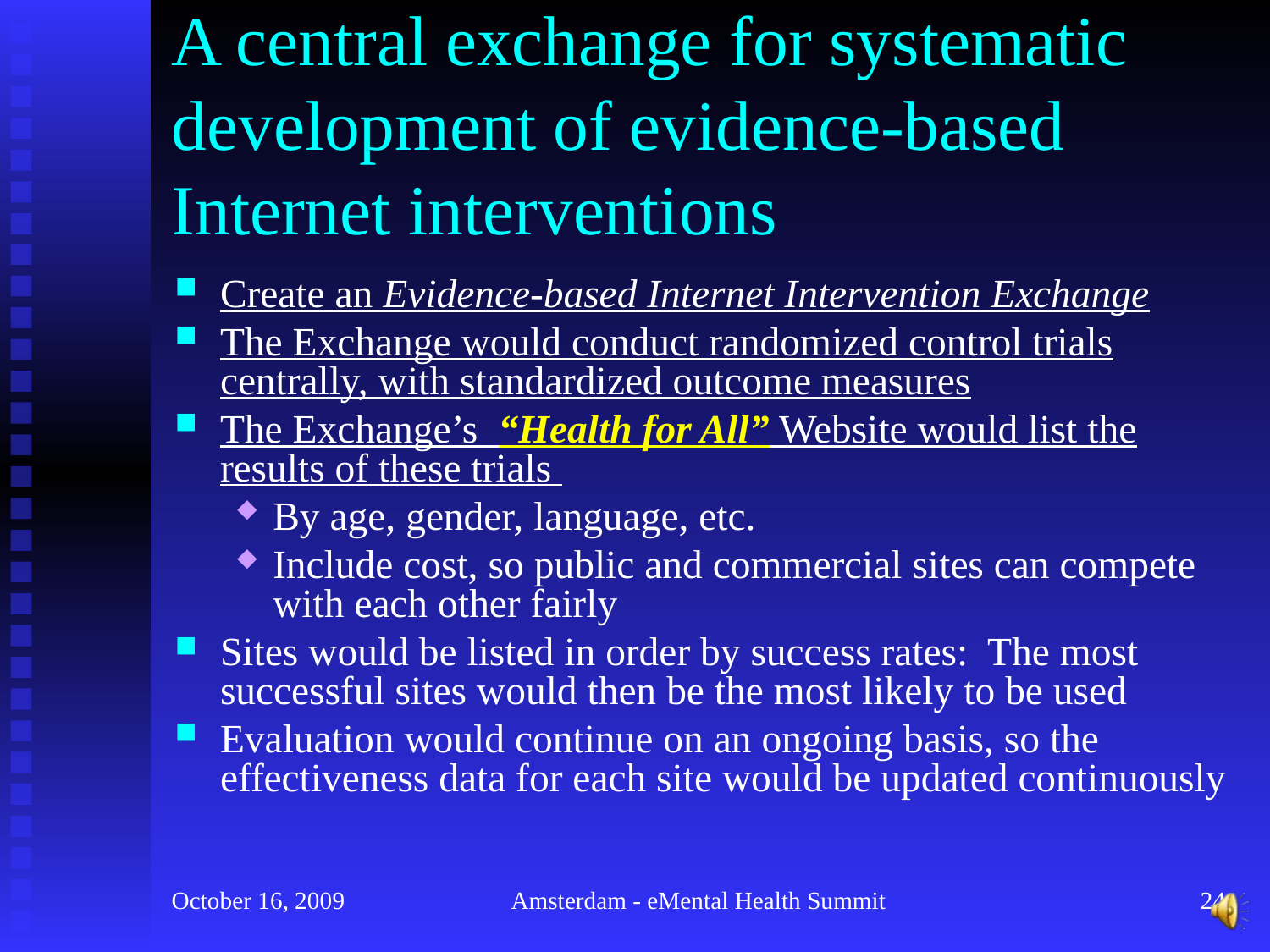

# A central exchange for systematic development of evidence-based Internet interventions
Create an Evidence-based Internet Intervention Exchange
The Exchange would conduct randomized control trials centrally, with standardized outcome measures
The Exchange’s “Health for All” Website would list the results of these trials
By age, gender, language, etc.
Include cost, so public and commercial sites can compete with each other fairly
Sites would be listed in order by success rates: The most successful sites would then be the most likely to be used
Evaluation would continue on an ongoing basis, so the effectiveness data for each site would be updated continuously
October 16, 2009
Amsterdam - eMental Health Summit
24

## Slide 25
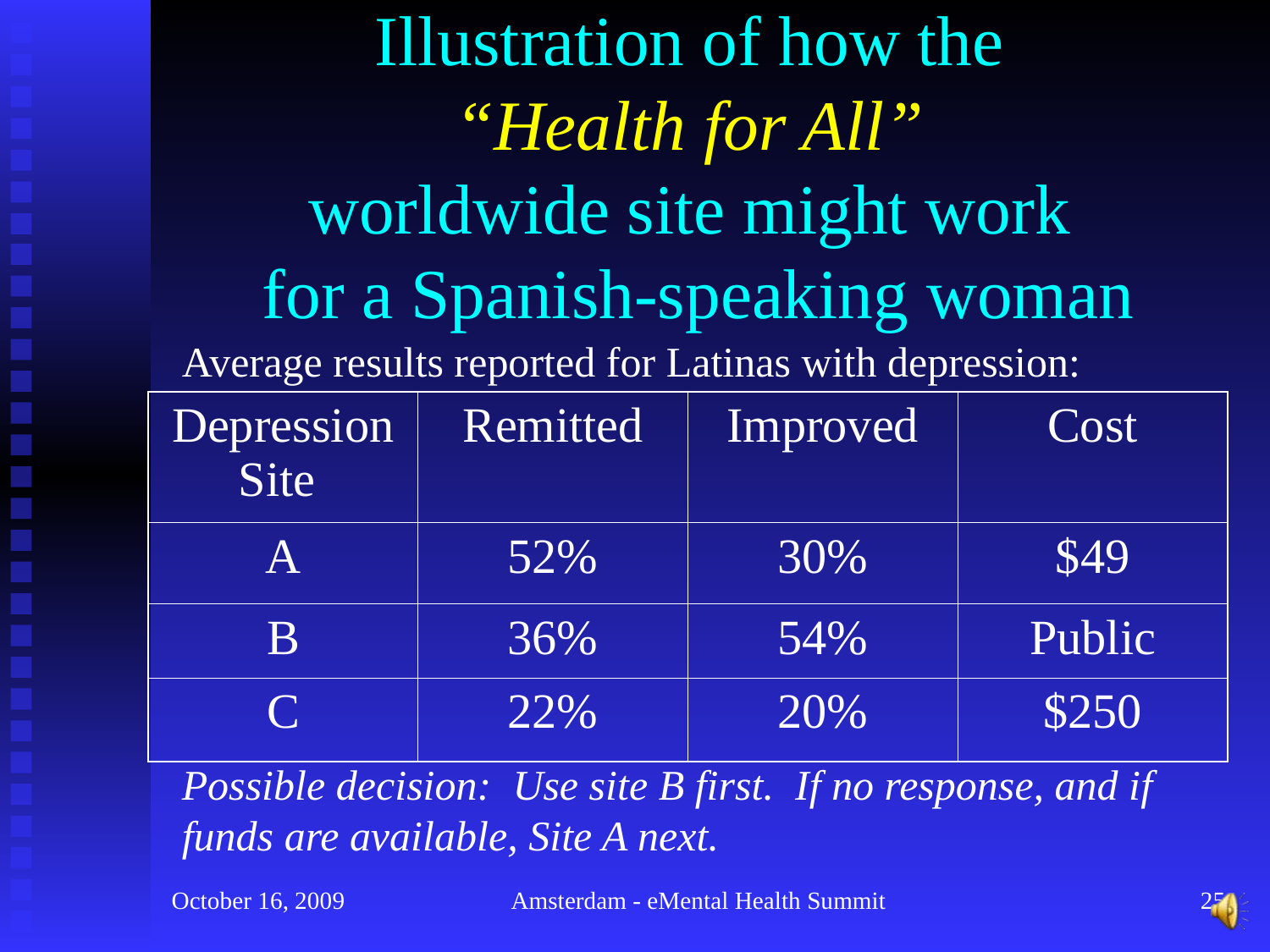

# Illustration of how the “Health for All” worldwide site might work for a Spanish-speaking woman
Average results reported for Latinas with depression:
| DepressionSite | Remitted | Improved | Cost |
| --- | --- | --- | --- |
| A | 52% | 30% | $49 |
| B | 36% | 54% | Public |
| C | 22% | 20% | $250 |
Possible decision: Use site B first. If no response, and if funds are available, Site A next.
October 16, 2009
Amsterdam - eMental Health Summit
25

## Slide 26
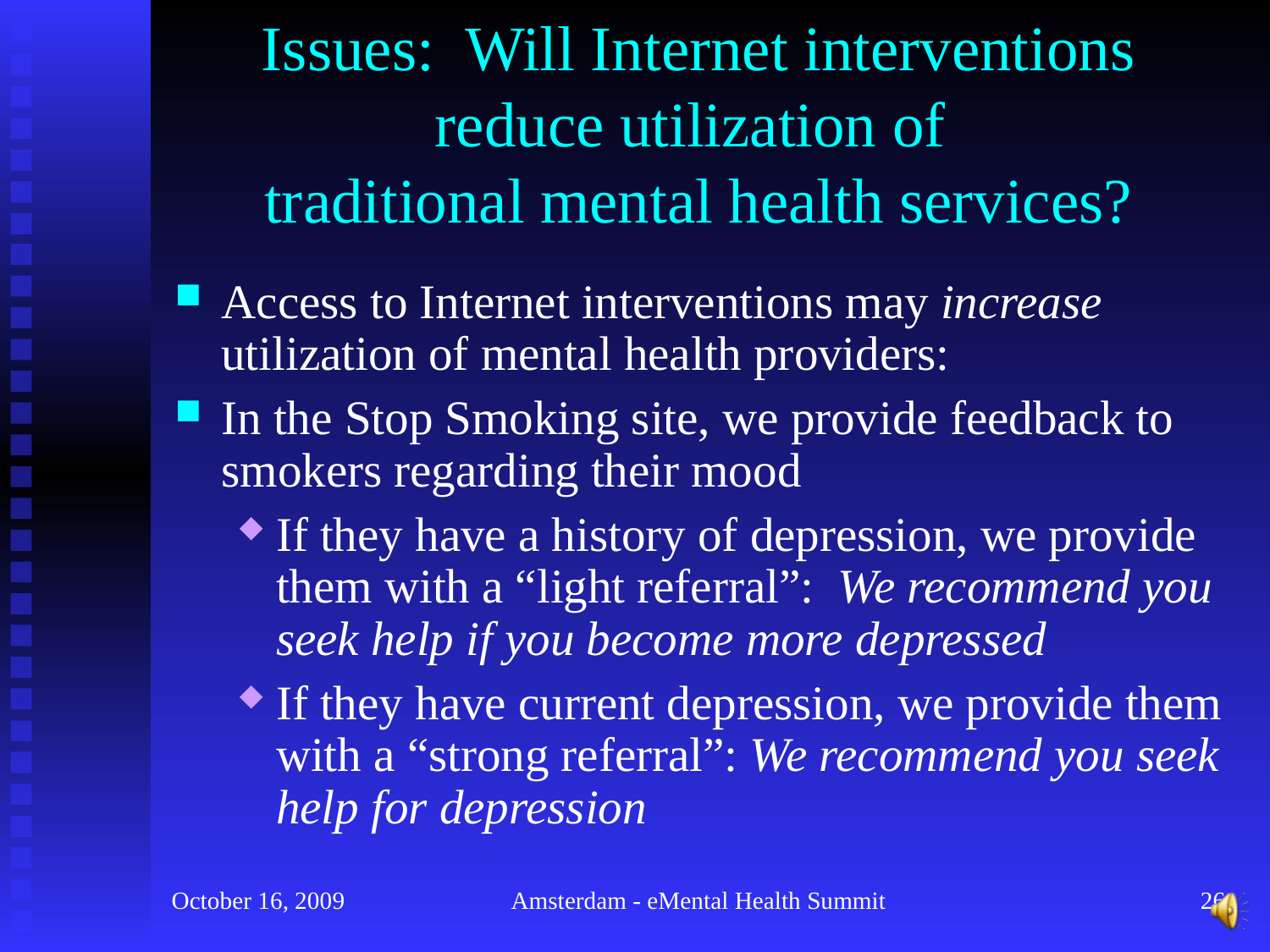

# Issues: Will Internet interventions reduce utilization of traditional mental health services?
Access to Internet interventions may increase utilization of mental health providers:
In the Stop Smoking site, we provide feedback to smokers regarding their mood
If they have a history of depression, we provide them with a “light referral”: We recommend you seek help if you become more depressed
If they have current depression, we provide them with a “strong referral”: We recommend you seek help for depression
October 16, 2009
Amsterdam - eMental Health Summit
26

## Slide 27
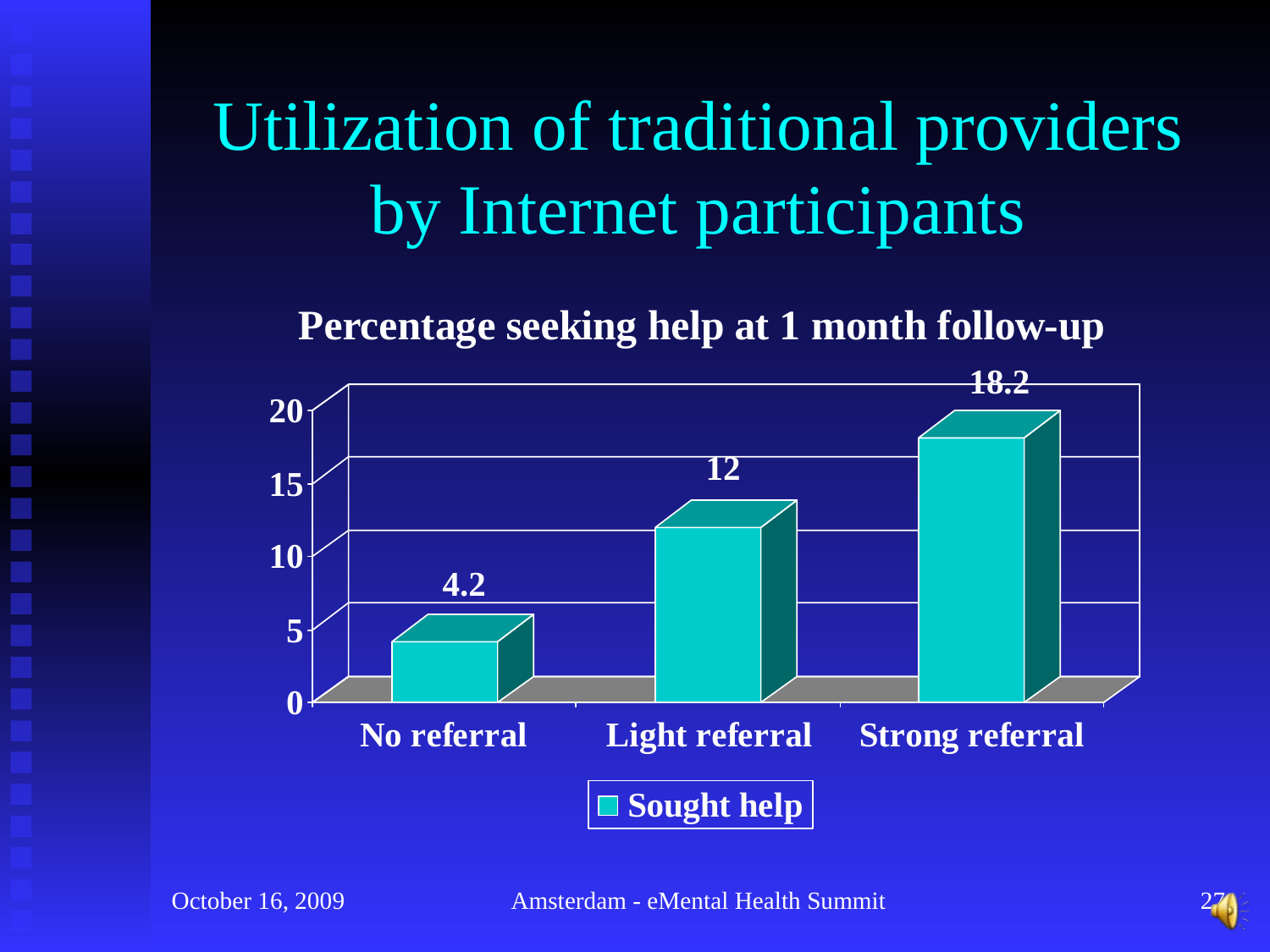

# Utilization of traditional providers by Internet participants
October 16, 2009
Amsterdam - eMental Health Summit
27

## Slide 28
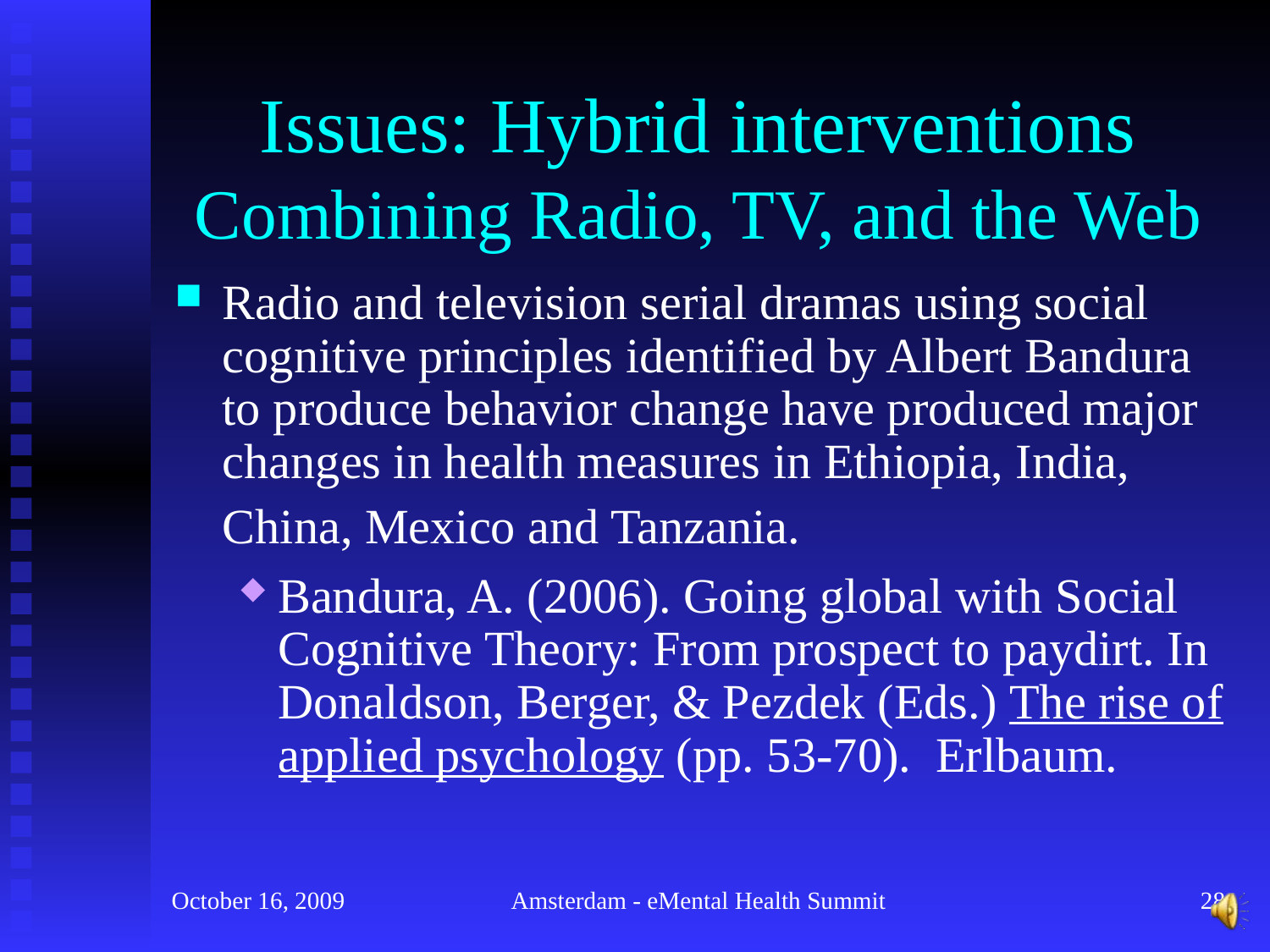

# Issues: Hybrid interventions Combining Radio, TV, and the Web
Radio and television serial dramas using social cognitive principles identified by Albert Bandura to produce behavior change have produced major changes in health measures in Ethiopia, India, China, Mexico and Tanzania.
Bandura, A. (2006). Going global with Social Cognitive Theory: From prospect to paydirt. In Donaldson, Berger, & Pezdek (Eds.) The rise of applied psychology (pp. 53-70). Erlbaum.
October 16, 2009
Amsterdam - eMental Health Summit
28

## Slide 29
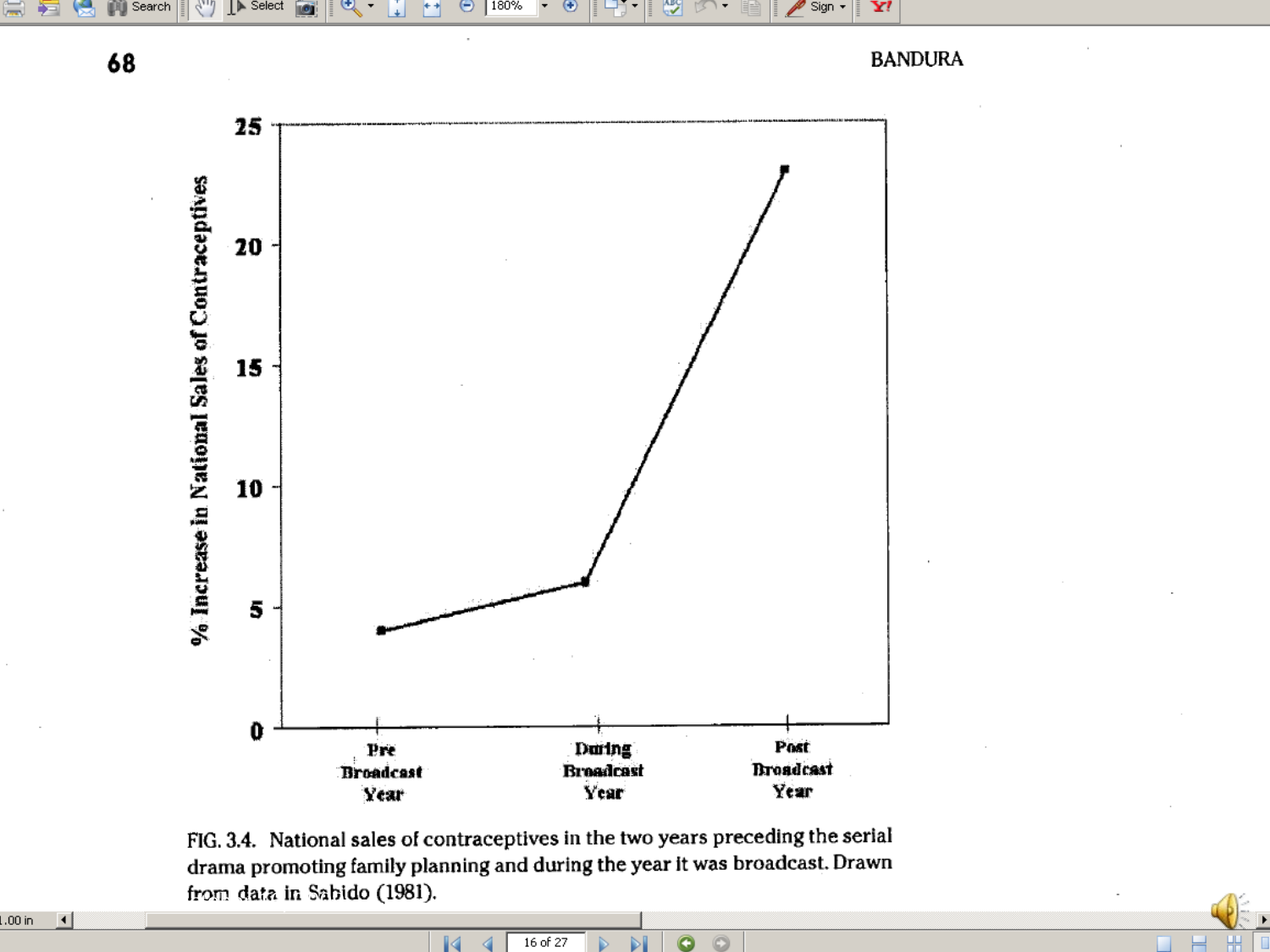

October 16, 2009
Amsterdam - eMental Health Summit
29

## Slide 30
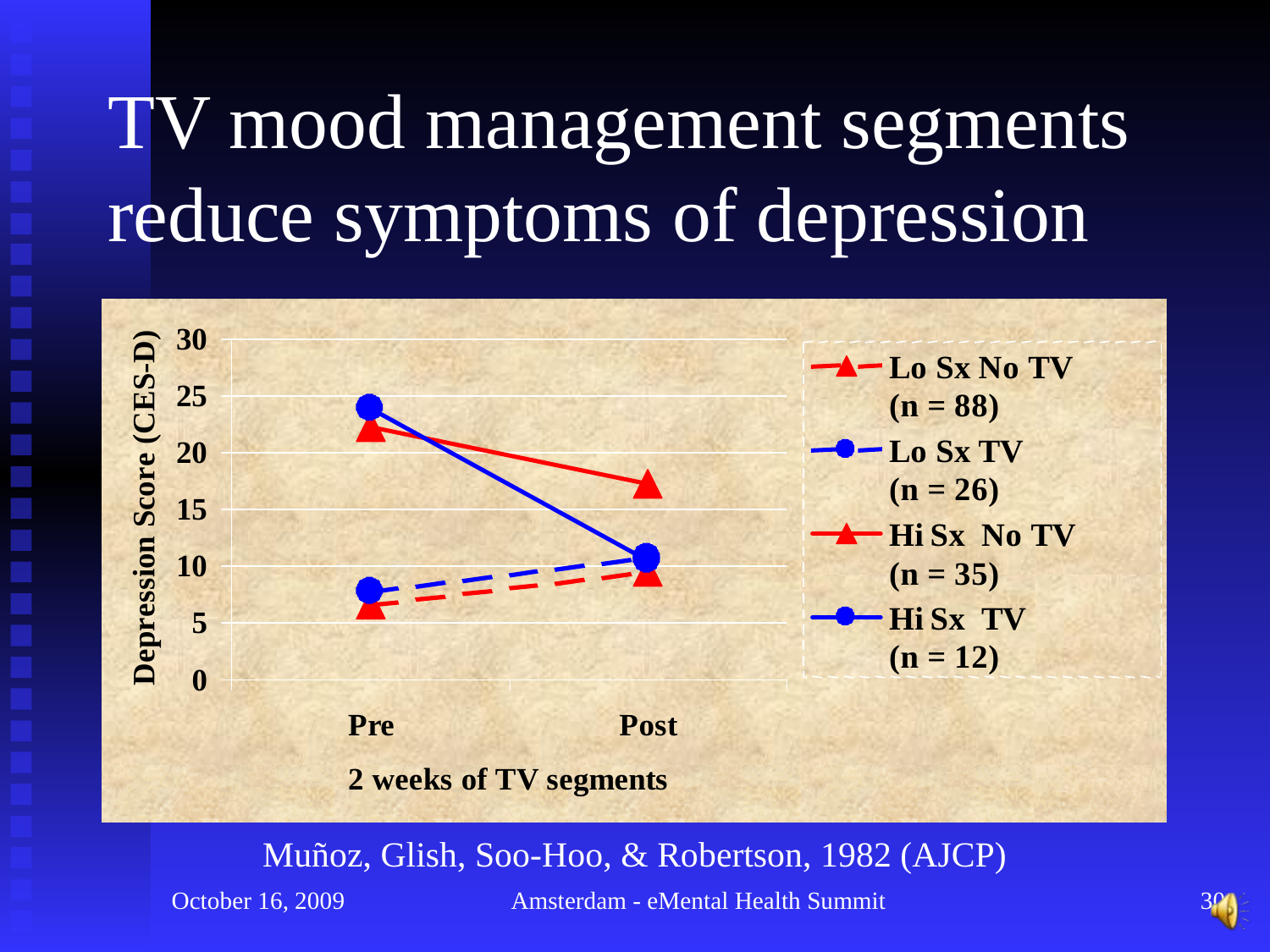

TV mood management segments reduce symptoms of depression
Muñoz, Glish, Soo-Hoo, & Robertson, 1982 (AJCP)
October 16, 2009
Amsterdam - eMental Health Summit
30

## Slide 31
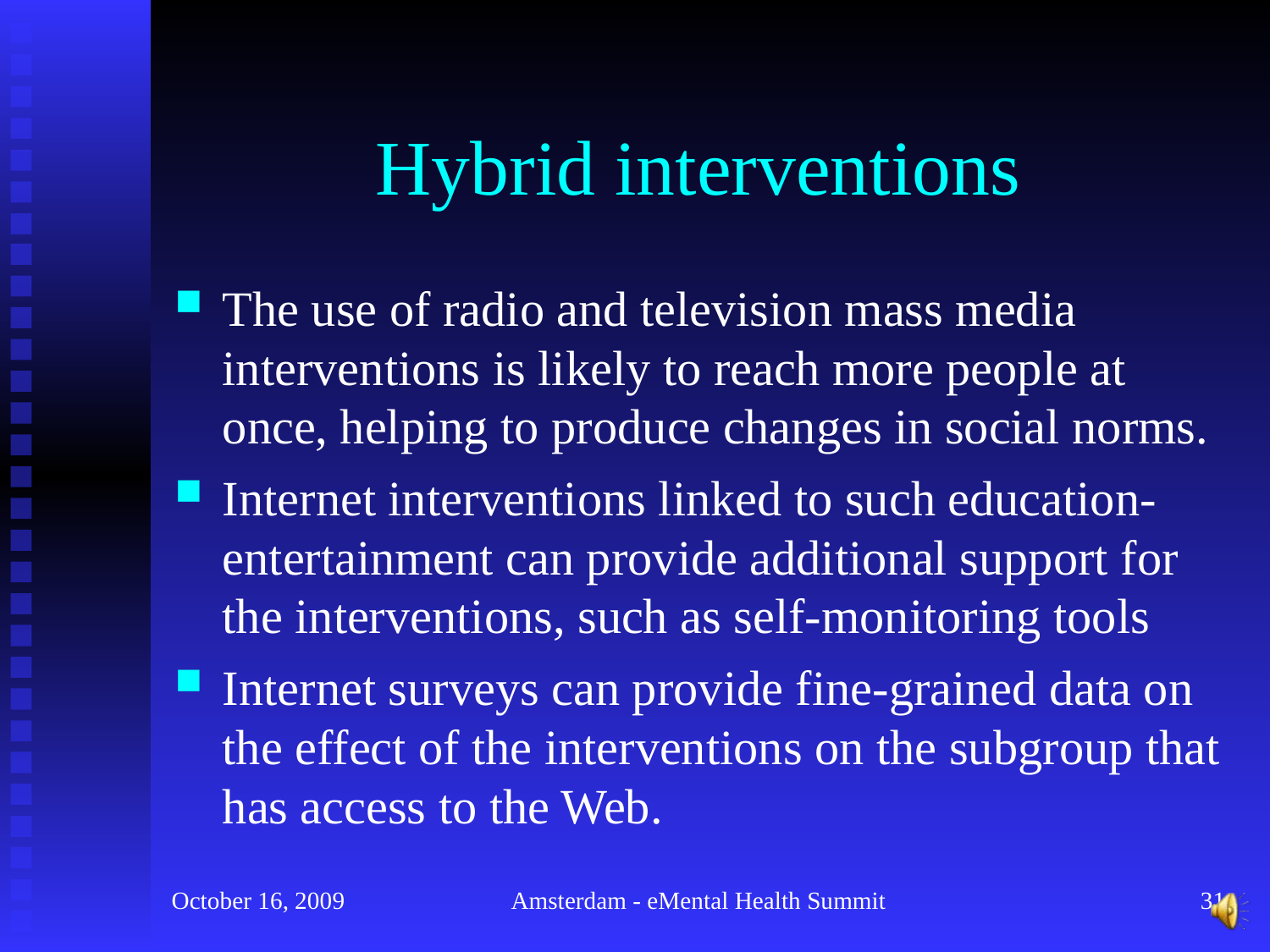

# Hybrid interventions
The use of radio and television mass media interventions is likely to reach more people at once, helping to produce changes in social norms.
Internet interventions linked to such education-entertainment can provide additional support for the interventions, such as self-monitoring tools
Internet surveys can provide fine-grained data on the effect of the interventions on the subgroup that has access to the Web.
October 16, 2009
Amsterdam - eMental Health Summit
31

## Slide 32
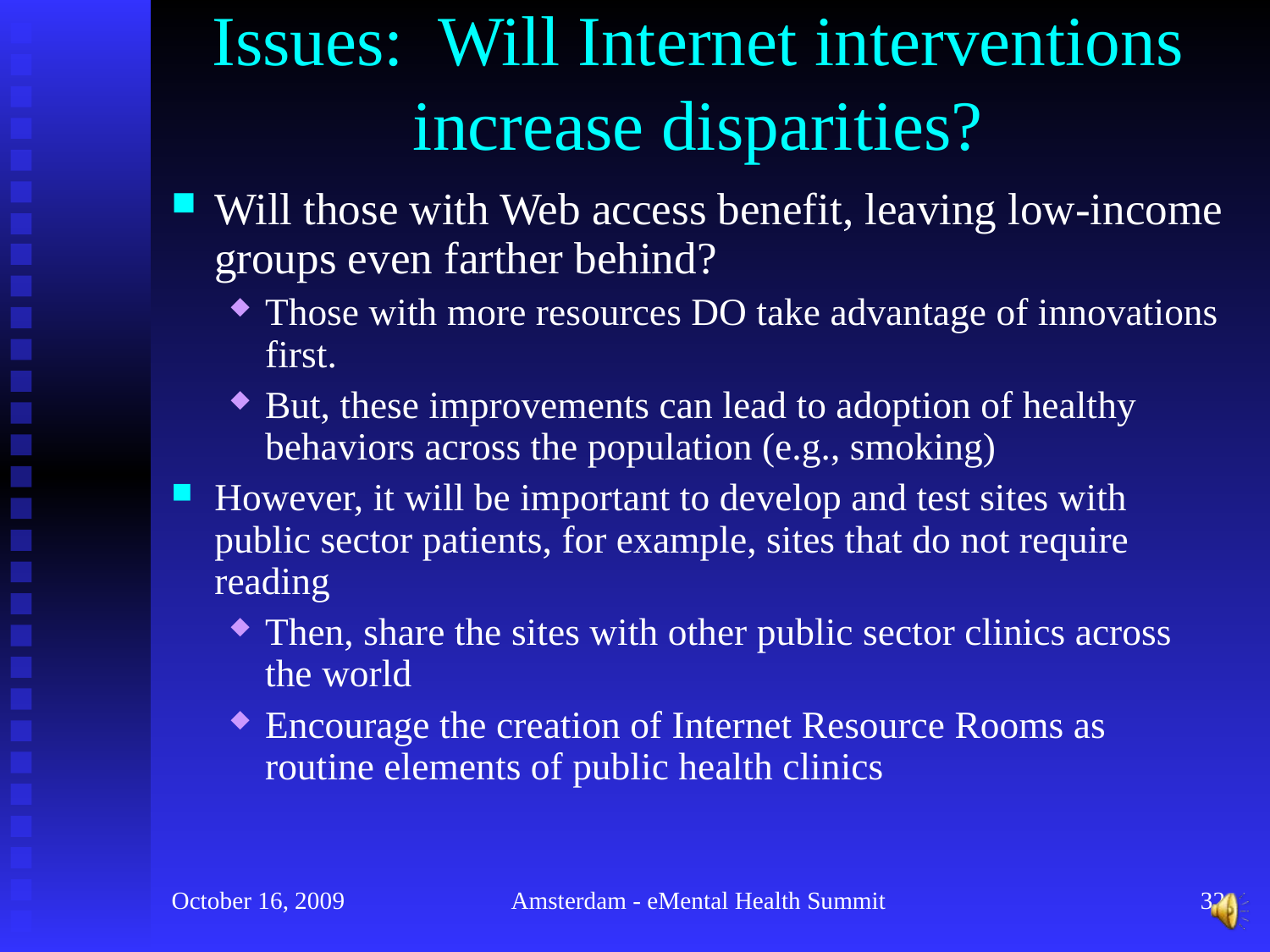

# Issues: Will Internet interventions increase disparities?
Will those with Web access benefit, leaving low-income groups even farther behind?
Those with more resources DO take advantage of innovations first.
But, these improvements can lead to adoption of healthy behaviors across the population (e.g., smoking)
However, it will be important to develop and test sites with public sector patients, for example, sites that do not require reading
Then, share the sites with other public sector clinics across the world
Encourage the creation of Internet Resource Rooms as routine elements of public health clinics
October 16, 2009
Amsterdam - eMental Health Summit
32

## Slide 33
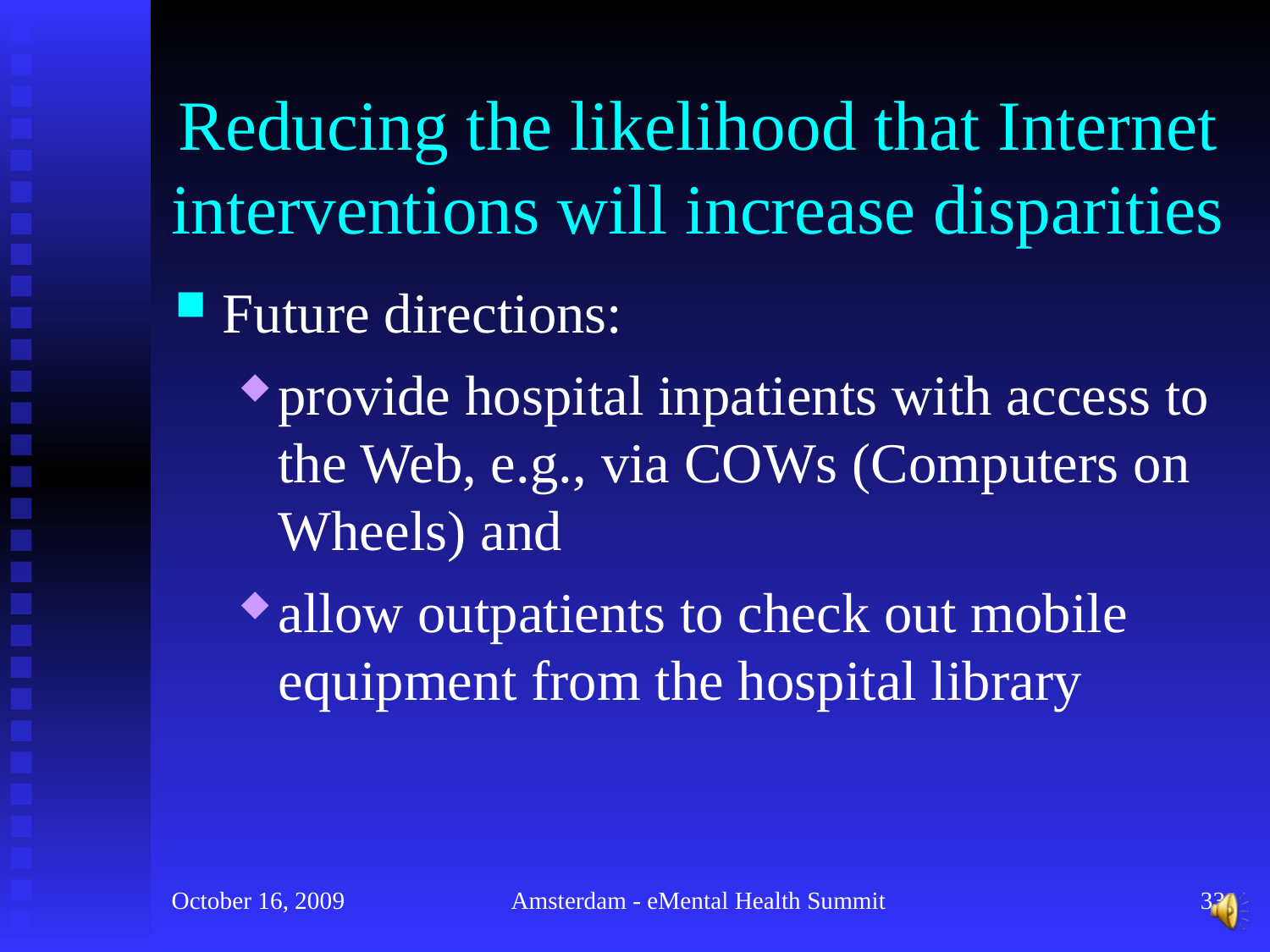

# Reducing the likelihood that Internet interventions will increase disparities
Future directions:
provide hospital inpatients with access to the Web, e.g., via COWs (Computers on Wheels) and
allow outpatients to check out mobile equipment from the hospital library
October 16, 2009
Amsterdam - eMental Health Summit
33

## Slide 34
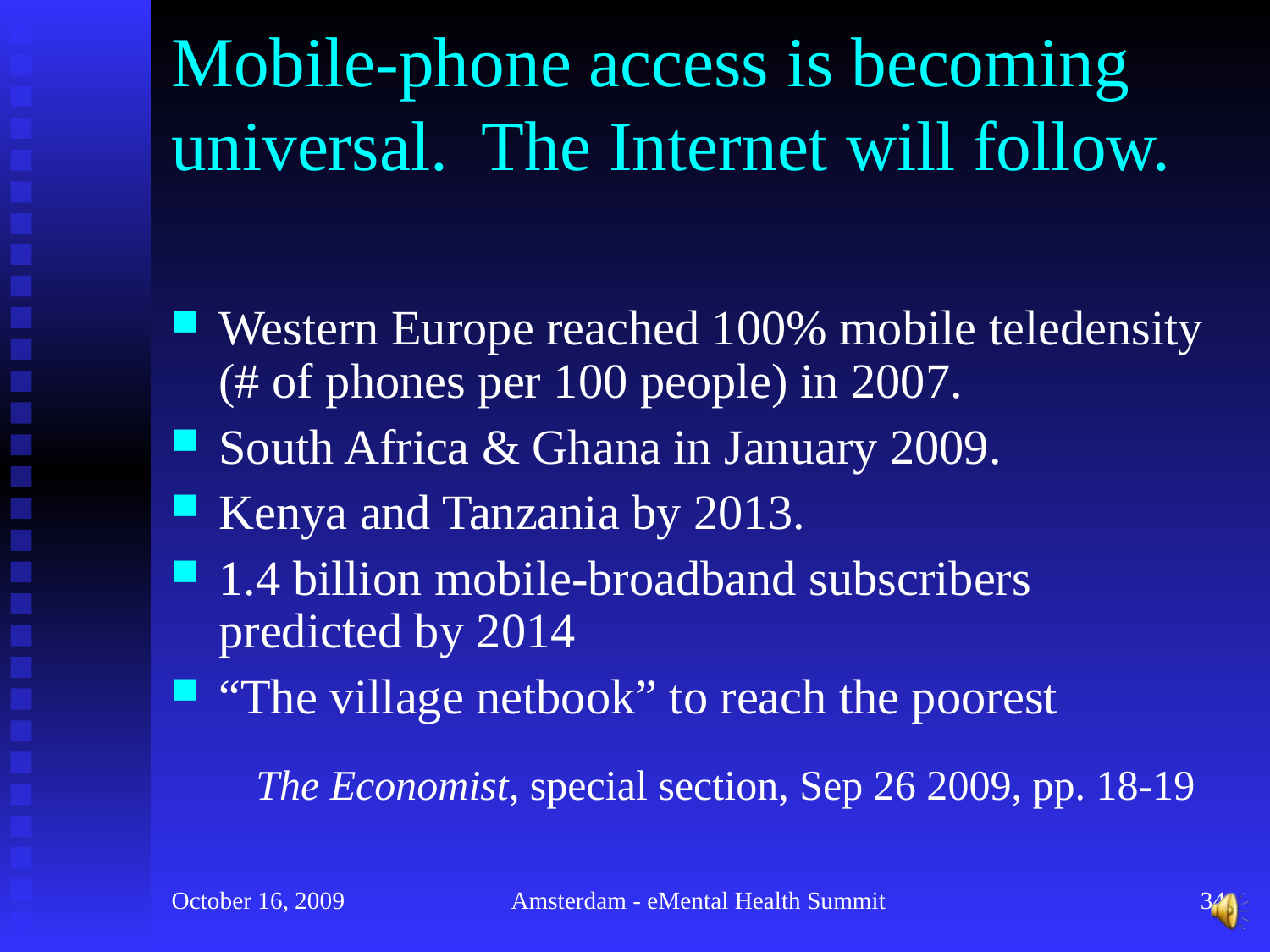

# Mobile-phone access is becoming universal. The Internet will follow.
Western Europe reached 100% mobile teledensity (# of phones per 100 people) in 2007.
South Africa & Ghana in January 2009.
Kenya and Tanzania by 2013.
1.4 billion mobile-broadband subscribers predicted by 2014
“The village netbook” to reach the poorest
The Economist, special section, Sep 26 2009, pp. 18-19
October 16, 2009
Amsterdam - eMental Health Summit
34

## Slide 35
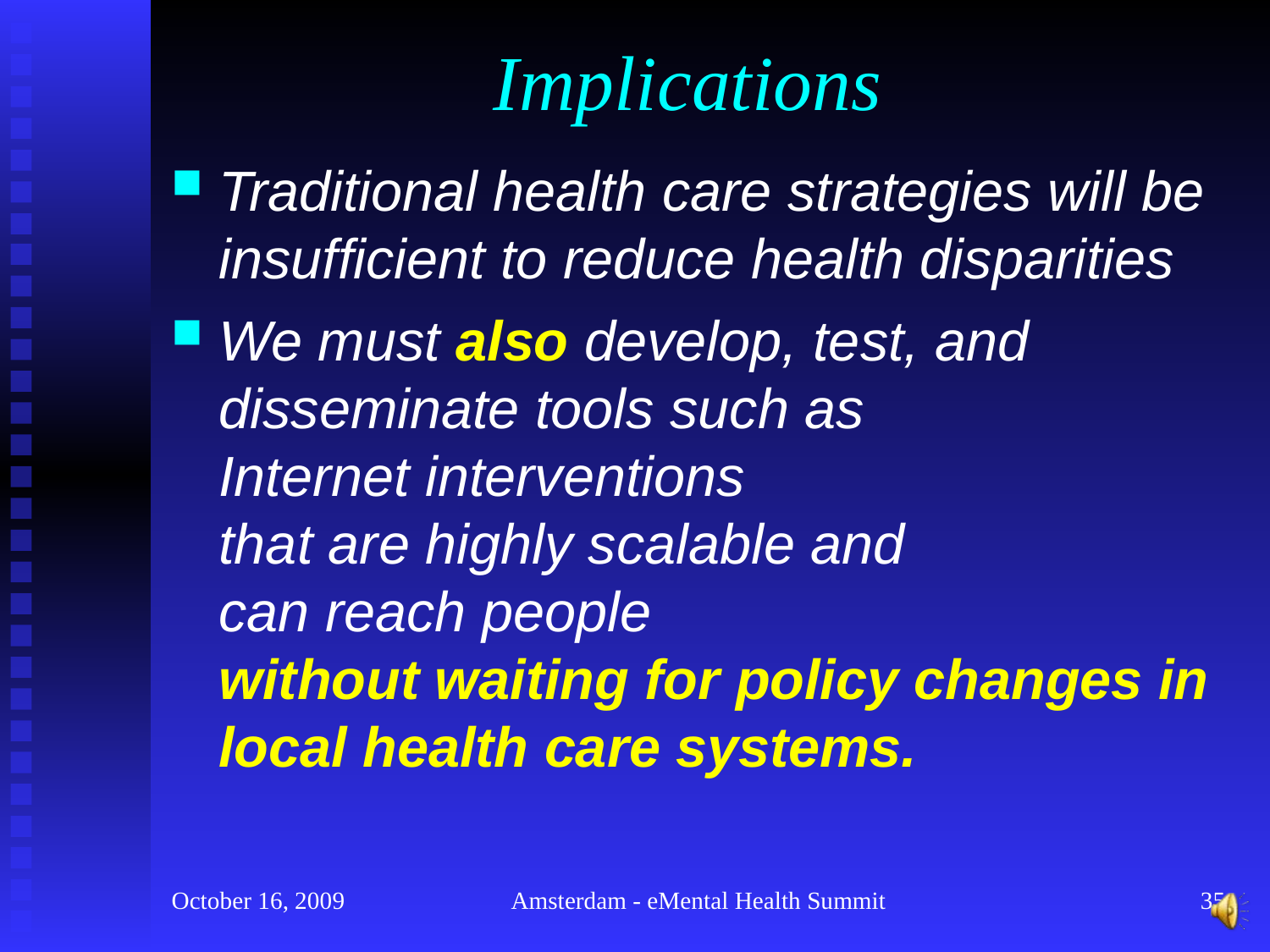

# Implications
Traditional health care strategies will be insufficient to reduce health disparities
We must also develop, test, and disseminate tools such as Internet interventions that are highly scalable and can reach people without waiting for policy changes in local health care systems.
October 16, 2009
Amsterdam - eMental Health Summit
35

## Slide 36
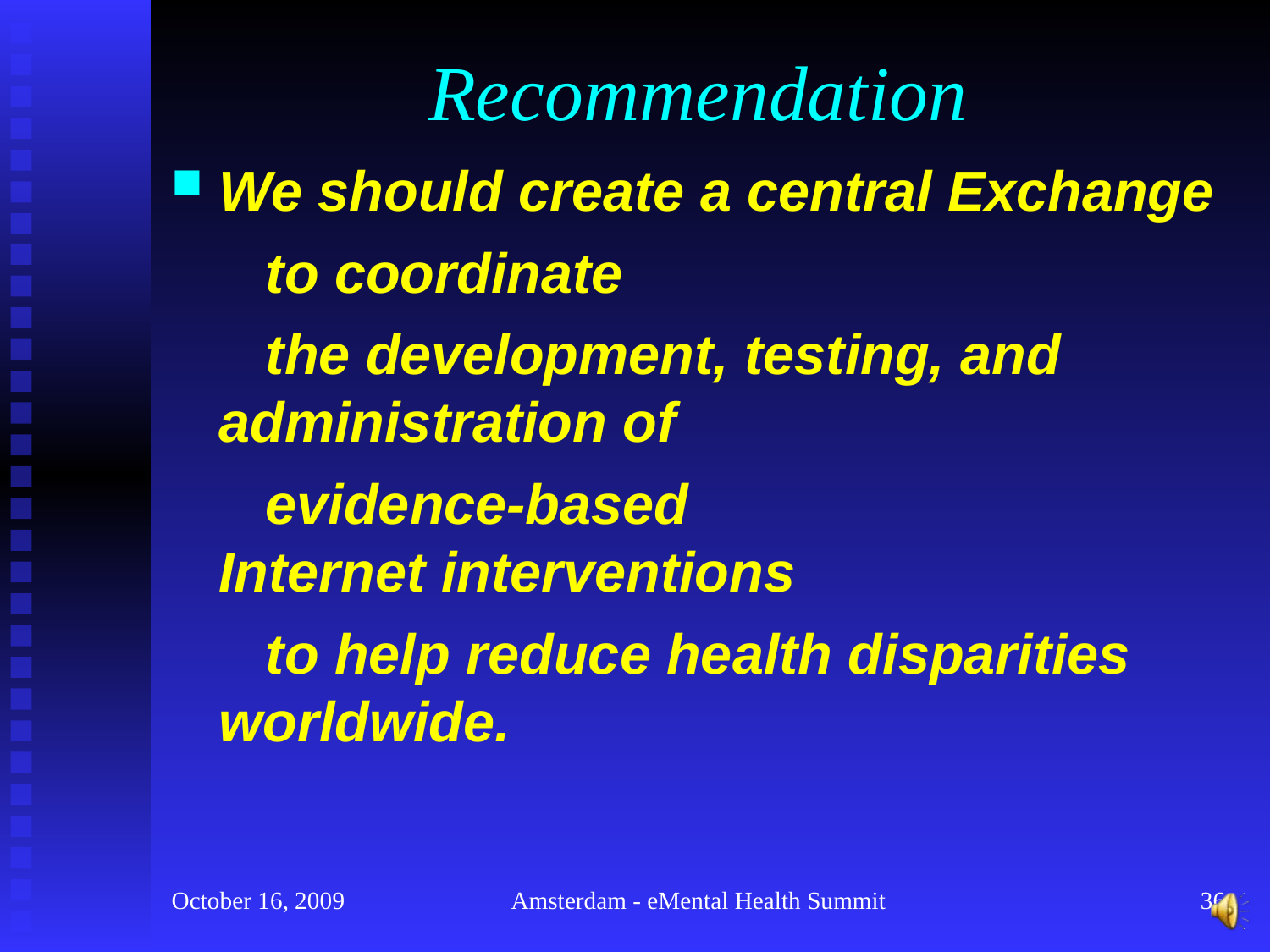

# Recommendation
We should create a central Exchange
 to coordinate
 the development, testing, and administration of
 evidence-based Internet interventions
 to help reduce health disparities worldwide.
October 16, 2009
Amsterdam - eMental Health Summit
36

## Slide 37
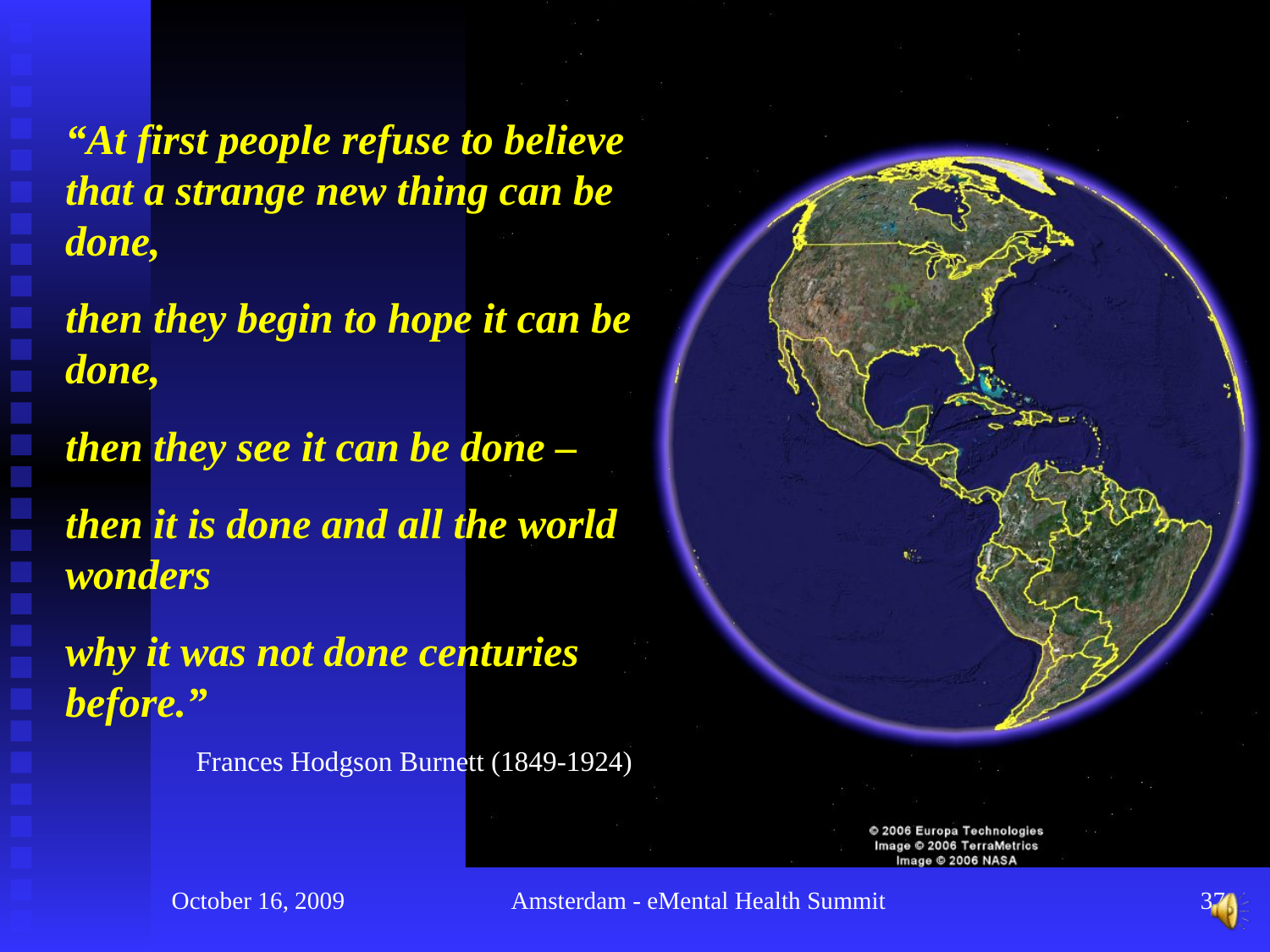

“At first people refuse to believe that a strange new thing can be done,
then they begin to hope it can be done,
then they see it can be done –
then it is done and all the world wonders
why it was not done centuries before.”
Frances Hodgson Burnett (1849-1924)
October 16, 2009
Amsterdam - eMental Health Summit
37

## Slide 38
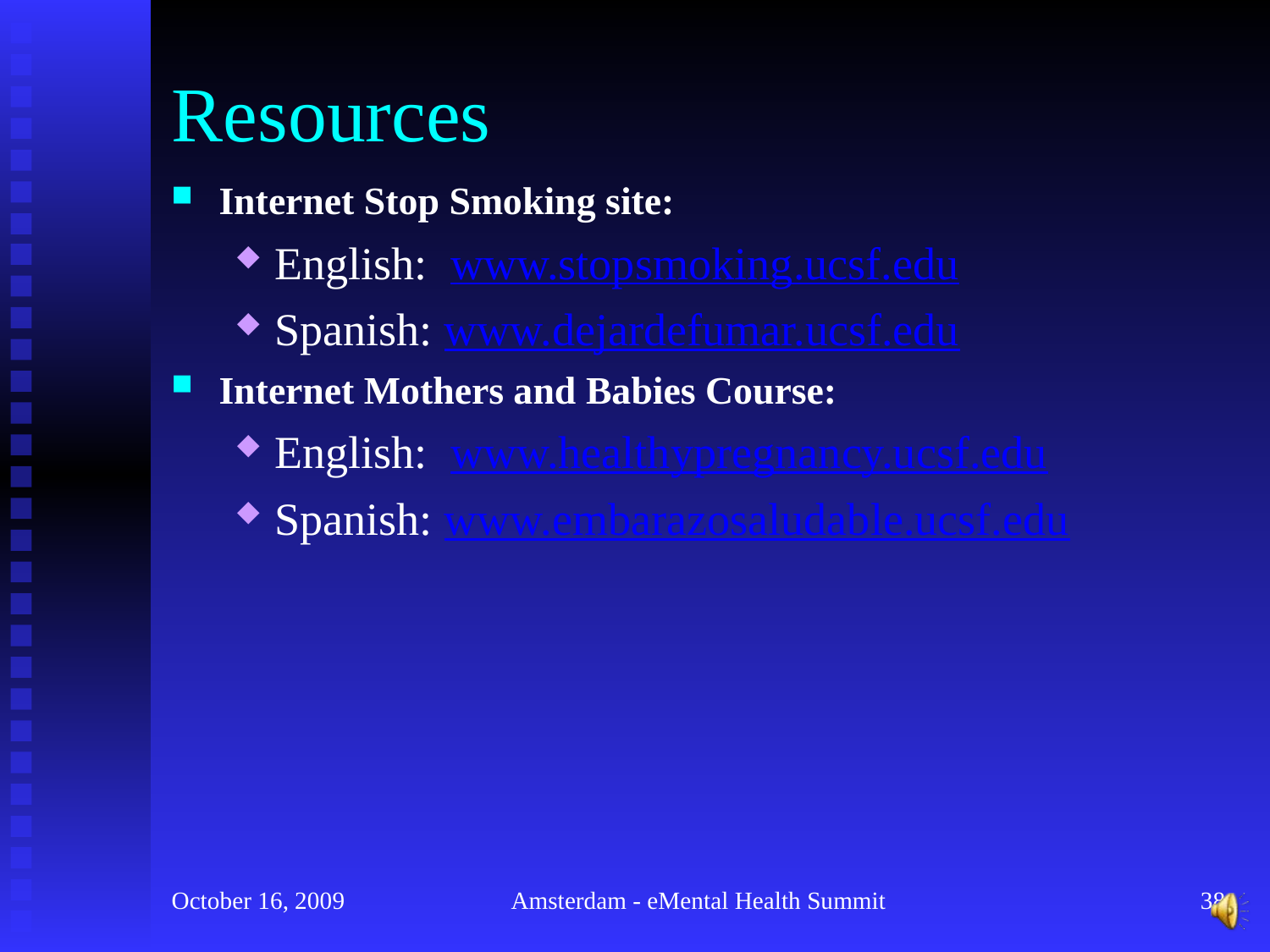

# Resources
Internet Stop Smoking site:
English: www.stopsmoking.ucsf.edu
Spanish: www.dejardefumar.ucsf.edu
Internet Mothers and Babies Course:
English: www.healthypregnancy.ucsf.edu
Spanish: www.embarazosaludable.ucsf.edu
October 16, 2009
Amsterdam - eMental Health Summit
38

## Slide 39
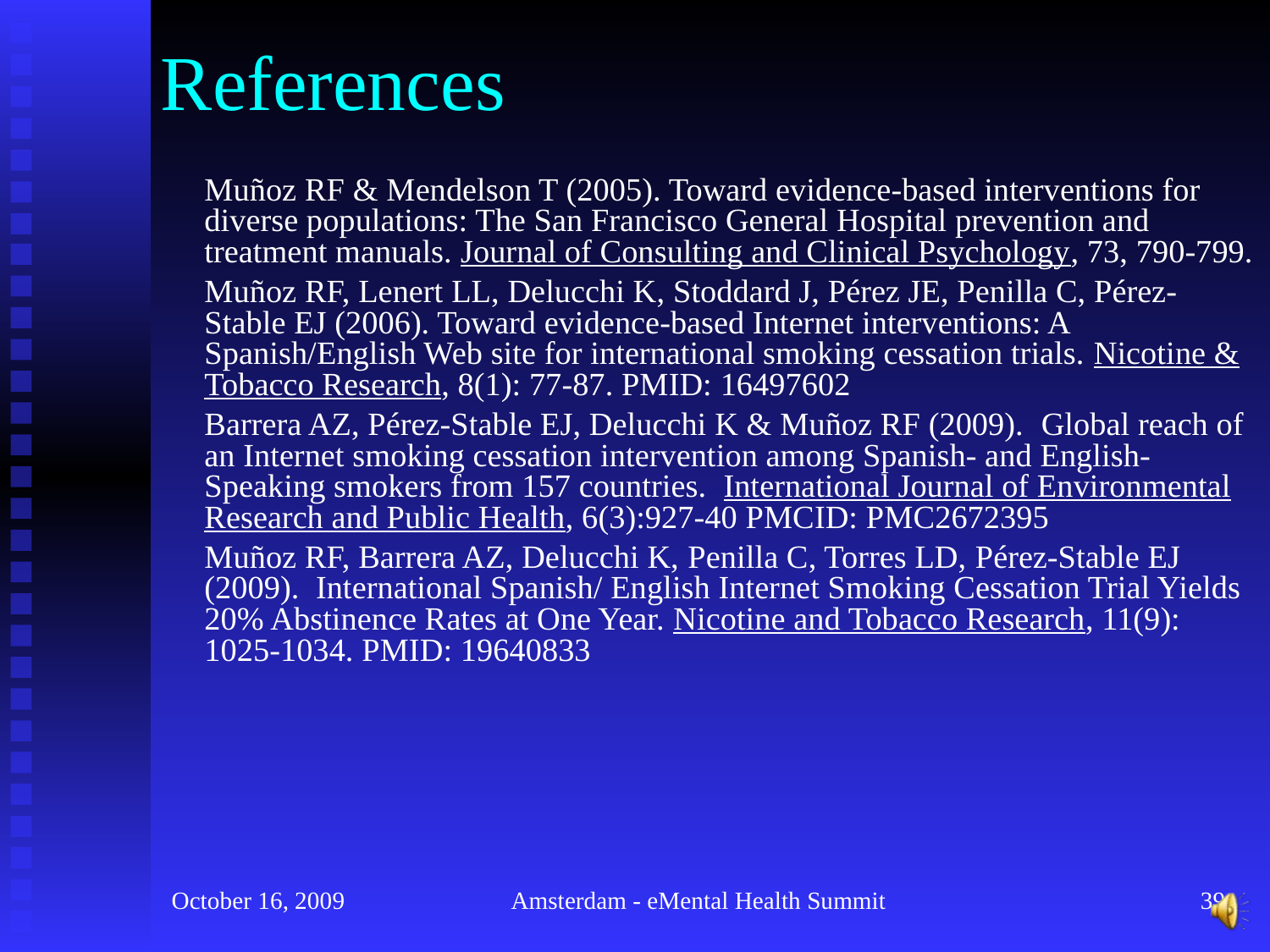

# References
Muñoz RF & Mendelson T (2005). Toward evidence-based interventions for diverse populations: The San Francisco General Hospital prevention and treatment manuals. Journal of Consulting and Clinical Psychology, 73, 790-799.
Muñoz RF, Lenert LL, Delucchi K, Stoddard J, Pérez JE, Penilla C, Pérez-Stable EJ (2006). Toward evidence-based Internet interventions: A Spanish/English Web site for international smoking cessation trials. Nicotine & Tobacco Research, 8(1): 77-87. PMID: 16497602
Barrera AZ, Pérez-Stable EJ, Delucchi K & Muñoz RF (2009). Global reach of an Internet smoking cessation intervention among Spanish- and English-Speaking smokers from 157 countries. International Journal of Environmental Research and Public Health, 6(3):927-40 PMCID: PMC2672395
Muñoz RF, Barrera AZ, Delucchi K, Penilla C, Torres LD, Pérez-Stable EJ (2009). International Spanish/ English Internet Smoking Cessation Trial Yields 20% Abstinence Rates at One Year. Nicotine and Tobacco Research, 11(9): 1025-1034. PMID: 19640833
October 16, 2009
Amsterdam - eMental Health Summit
39
